# Critical Episodes of Inflammation, Catabolism, and Immunosuppression Define Mortality Risk in Chronic Critical Illness

**DOI:** 10.3390/jcm15207864

**Published:** 2026-10-12

**Authors:** Valery Likhvantsev, Levan Berikashvili, Petr Polyakov, Mikhail Yadgarov, Alexey Yakovlev, Andrey Grechko

**Affiliations:** Federal Research and Clinical Center of Intensive Care Medicine and Rehabilitology, Moscow 107031, Russia; lberikashvili@fnkcrr.ru (L.B.); p.polyakov@fnkcrr.ru (P.P.); myadgarov@fnkcrr.ru (M.Y.); ayakovlev@fnkcrr.ru (A.Y.); fnkcrr@fnkcrr.ru (A.G.)

**Keywords:** chronic critical illness, persistent inflammation, immunosuppression and catabolism syndrome, mortality, PICS

## Abstract

**Background/Objectives**: A globally recognized definition of chronic critical illness (CCI) is lacking. Temporal criteria produce variable estimates, while pathophysiological models emphasize inflammation, catabolism, and immunosuppression (ICS). The relationship between ICS dynamics and mortality remains unclear. **Methods**: This retrospective single-center cohort study analyzed real-world data from 3374 adult intensive care unit (ICU) patients. CCI was defined by first detection of the ICS triad: C-reactive protein (CRP) >20 mg/L, albumin <30 g/L, and absolute lymphocyte count <0.8 × 10^9^/L within 24 h. CCI days were classified as critical or non-critical. Mortality and mortality density were compared across CCI groups and periods. **Results**: CCI developed in 820 patients (24.3%) and was associated with higher mortality (relative risk (RR) 5.08) and mortality density (incidence rate ratio (IRR) 4.09). Critical periods comprised 22.15% of CCI patient-days and had higher mortality density than non-critical periods (IRR 4.25) and no CCI (IRR 10.10). **Conclusions**: CCI appears to be a biologically dynamic condition characterized by alternating critical and non-critical periods. Periods of simultaneous inflammation, catabolism, and immunosuppression are associated with the highest mortality density, while elevated mortality persists even during periods of partial biological compensation. These findings support the concept that the current ICS state may reflect clinically relevant changes in the course of CCI. However, they do not establish diagnostic or predictive performance and require external validation.

## 1. Introduction

Despite its clinical importance, a universally accepted definition of chronic critical illness (CCI) has not yet been established. Earlier proposals relied on arbitrary time-based criteria, such as a minimum length of intensive care unit (ICU) stay, a specified duration of mechanical ventilation, or the presence of a tracheostomy [1,2,3,4]. This approach, however, has led to wide variability in reported CCI prevalence, ranging from 3% to 55% depending on the definition used [5]. Such heterogeneity makes it impossible to compare study populations or to develop consistent prevention and treatment strategies [6]. Reported outcomes are similarly heterogeneous, with mortality varying from less than 10% to more than 60% across CCI cohorts, reflecting differences in patient populations, underlying diagnoses, illness severity, and the criteria used to define CCI [5].

Strictly time-based definitions of chronic critical illness appear to have limited conceptual and clinical utility [5,6]. Greater insight may be gained from identifying the characteristic physiological pathways driving CCI development or the distinctive clinical features that define the syndrome. A central concept in the first area is the triad of persistent inflammation, immunosuppression, and catabolism, widely referred to as PICS (persistent inflammation, immunosuppression, and catabolism syndrome) [7]. These processes are closely interconnected. Persistent inflammation reflects sustained activation of the inflammatory response, catabolism contributes to progressive loss of protein and muscle mass, while immunosuppression increases susceptibility to secondary infections and may impair recovery. Their coexistence may therefore represent a particularly unfavorable biological state during prolonged critical illness [7]. In its classic formulation, PICS includes not only the three physiological components but also a mandatory time criterion, typically an ICU stay exceeding 10 days [7]. However, accumulating evidence suggests that the temporal threshold itself may not be the key determinant of poor outcomes [8]. Our previous work has shown that the simultaneous presence of all three components (inflammation, catabolism and immunosuppression; the ICS triad) at any point during the ICU course is independently associated with increased mortality, regardless of when it first appears [8]. These findings do not imply that time is irrelevant to the definition of CCI. Rather, CCI should be considered only after the acute phase of the underlying disease has passed. The duration of the acute phase varies across diseases and clinical trajectories, making a universal threshold based solely on the number of ICU days potentially inappropriate. We therefore propose that, once the acute phase has resolved, the first documented occurrence of the ICS triad may provide a biologically defined marker of CCI formation. In addition, other pathophysiological features of prolonged ICU stay that may affect hospital outcomes are currently being actively investigated [9,10].

Building on these observations, we have proposed that the three components of ICS may not always move in synchrony. Instead, periods during which all three elements are present simultaneously (critical periods) may alternate with intervals in which at least one component is temporarily absent or compensated (non-critical periods). This concept of asynchronous dynamics has not been previously examined in relation to patient outcomes. In particular, it remains unknown whether mortality differs between critical and non-critical periods of CCI, and how both compare with patients without CCI. The present study was not intended to determine whether time can be eliminated from the definition of CCI, but rather to examine the biological heterogeneity of CCI after its development. We therefore hypothesized that, once CCI has developed, its course is not biologically homogeneous and that mortality is higher during periods when all three ICS components are simultaneously present than during periods in which at least one component is absent.

Accordingly, the objective of this study was to compare mortality among patients without CCI, all patients with CCI, and patients with CCI during critical versus non-critical periods.

## 2. Materials and Methods

### 2.1. Source of Data

The Federal Research and Clinical Center of Intensive Care Medicine and Rehabilitology (FRCC ICMR) is one of the largest tertiary centers in the Russian Federation, specializing in the management and research of prolonged critical illness and chronic critical illness (PCI/CCI). The primary data source for this study was the institutional Russian Intensive Care Dataset (RICD) v3.0. [11]. The anonymized dataset comprised 8135 patients admitted to the FRCC ICMR, of whom 4050 had intensive care unit (ICU) admissions.

### 2.2. Study Design and Setting

A retrospective single-center cohort study was conducted using real-world clinical data from the RICD v3.0. [11].

For the purpose of this study, we screened all ICU admissions recorded in the RICD dataset between December 2017 and September 2025. The following inclusion criteria were applied: (1) first admission to the ICU; (2) age over 18 years; (3) the availability of at least one evaluable assessment of ICS status. The diagnostic criteria for ICS were: C-reactive protein >20 mg/L, albumin <30 g/L, and an absolute lymphocyte count <0.8 × 10^9^/L, recorded simultaneously within a 24-h period [8]. The exclusion criteria were: (1) ICU length of stay <24 h; (2) absence of documented International Classification of Diseases, 10th Revision (ICD-10) diagnosis records.

All eligible cases from the dataset were included; therefore, no formal sample size calculation was performed. The study used de-identified data from the RICD v3.0, for which the anonymization algorithm had been reviewed and approved by the Local Ethics Committee of the Federal Research and Clinical Center of Intensive Care Medicine and Rehabilitology (Committee decision No. 4/23/2 dated 20 December 2023). Accordingly, the Committee confirmed that separate formal ethical approval was not required for this retrospective study. The study was conducted in accordance with the Strengthening the Reporting of Observational Studies in Epidemiology (STROBE) statement [12]. The STROBE checklist is provided in Supplementary Table S1.

### 2.3. Data Management

For the present analysis, the following data were extracted from RICD version 3.0: clinical and demographic characteristics, primary diagnoses at admission, as well as information on comorbidities and medical history. Imputation of missing values was not applied.

Additionally, severity of illness was assessed using the Glasgow Coma Scale (GCS), Full Outline of UnResponsiveness (FOUR), Coma Recovery Scale–Revised (CRS-R), Disability Rating Scale (DRS), modified Rankin Scale (mRS), and Sequential Organ Failure Assessment 2 (SOFA-2), along with laboratory parameters including complete blood count, biochemical markers, and coagulation profile parameters. Hospital course characteristics were also taken into account (ICU mortality, nosocomial pneumonia, sepsis, multiple organ failure, need for mechanical ventilation and vasopressor support, length of ICU and total hospital stay). The onset of CCI was defined as the time of the first documented ICS episode. Over the course of chronic critical illness, days were divided into critical and non-critical periods. The onset of a critical CCI episode was defined as the day on which all three components of the ICS triad were simultaneously present. A critical CCI episode was considered resolved when laboratory test results indicated that at least one component no longer fulfilled the criteria for the ICS triad [8]. Patients were not permanently assigned to either state. If the complete ICS triad subsequently reappeared after resolution of a critical episode, a new critical episode was recorded. Thus, an individual patient could contribute multiple critical and non-critical periods during the CCI course. For the mortality-density analysis, all patient-days contributed by a patient were assigned to the corresponding current state, and deaths were attributed to the state in which they occurred.

Data extraction was performed using DB Browser for SQLite version 3.13.1.

### 2.4. Statistical Analysis

Normality of data distribution was assessed using the Shapiro–Wilk test. Continuous variables were expressed as median and interquartile range (IQR), while categorical variables were presented as absolute (*n*) and relative (%) frequencies. Between-group comparisons of ICS characteristics and hospitalization course features were performed using the nonparametric Mann–Whitney U test and the chi-square test, as appropriate for the data type. For categorical variables, odds ratios (OR) with 95% confidence intervals were additionally calculated for between-group comparisons. ORs were calculated from 2 × 2 contingency tables, and their 95% confidence intervals were calculated on the natural logarithmic scale using asymptotic standard errors and subsequently back-transformed by exponentiation. The *p*-values reported for categorical between-group comparisons were obtained using the chi-square test. Comparisons of baseline characteristics and laboratory variables were considered exploratory; no adjustment for multiple testing was applied for these analyses.

To evaluate mortality, two complementary analyses were performed. First, mortality frequency was calculated as the ratio of the number of deaths to the total number of patients in each group: patients without CCI, all patients with CCI, and, separately, deaths occurring during the non-critical and critical periods of CCI. Pairwise comparisons were performed using relative risk (RR) with 95% confidence intervals (CI). In addition, mortality density was assessed as the number of deaths per 1000 patient-days. This measure was calculated for the same groups, and between-group differences were evaluated using the incidence rate ratio (IRR) with 95% CI. In patients with CCI, patient-days and deaths were assigned to the non-critical or critical period according to the time of their occurrence. RRs were calculated as ratios of mortality proportions, whereas IRRs were calculated as ratios of mortality rates based on the corresponding numbers of deaths and accumulated patient-time. For RRs and IRRs, 95% CIs were calculated on the natural logarithmic scale using asymptotic standard errors and subsequently back-transformed by exponentiation. Corresponding *p*-values were obtained using asymptotic Wald tests of the null hypothesis that the respective ratio was equal to 1. All *p*-values were two-sided. Because four pairwise comparisons were performed for mortality frequency and six pairwise comparisons for mortality density, the corresponding Bonferroni-adjusted significance thresholds were *p* < 0.0125 and *p* < 0.0083, respectively.

All statistical analyses were performed using IBM SPSS Statistics for Windows (version 27.0, IBM Corp, Armonk, NY, USA). Visualization of ICS episodes was performed using a swimmer plot created with Python version 3.11.5 (numpy, pandas, and matplotlib libraries). During the preparation of this manuscript the authors used ChatGPT 5.3 in order to improve readability and language. After using this tool, the authors reviewed and edited the content as needed and take full responsibility for the content of the publication.

## 3. Results

### 3.1. Patient Characteristics

A total of 3374 patients met the eligibility criteria and were included in the analysis, of whom 820 (24.3%) had CCI and 2554 (75.7%) did not (Figure 1).

Compared to patients without CCI, those with CCI were older (68 (55–77) vs. 60 (45–71) years, *p* < 0.001), whereas body mass index (BMI) did not differ between the groups. Patients with CCI also had higher illness severity at admission, as indicated by lower GCS and FOUR scores and a higher SOFA-2 score (all *p* < 0.001). Additionally, pneumonia on admission was more frequent in the CCI group (57.8% vs. 38.3%, *p* < 0.001; OR 2.21, 95% CI 1.88–2.59). Among the major comorbidities, coronary artery disease, chronic heart failure, arterial hypertension, and chronic kidney disease were more prevalent in patients with CCI (Table 1).

Upon ICU admission, patients who later developed CCI had significantly higher white blood cell counts (9.6 vs. 8.9 × 10^9^/L, *p* < 0.001) and lower absolute lymphocyte counts (0.90 vs. 1.49 × 10^9^/L, *p* < 0.001), with a corresponding lower lymphocyte percentage (9.7% vs. 16.8%, *p* < 0.001). Total protein and albumin levels were also significantly lower in the CCI group (57.5 vs. 62.8 g/L and 28.2 vs. 32.8 g/L, respectively; both *p* < 0.001), whereas C-reactive protein was markedly higher (69.5 vs. 33.3 mg/L, *p* < 0.001) (Table 2).

Patients with CCI experienced significantly worse ICU outcomes compared to those without CCI. The ICU mortality rate was higher in the CCI group (29.6% vs. 5.8%; *p* < 0.001; OR 6.80, 95% CI 5.44–8.50). Furthermore, patients with CCI had higher rates of nosocomial pneumonia, sepsis, and multiple organ failure, and were more likely to require mechanical ventilation and vasopressor support (*p* < 0.001 for all). The CCI group also had a markedly longer ICU stay (30 (21–55) vs. 19 (7–29) days, *p* < 0.001), while total hospital length of stay did not differ significantly between the two groups (Table 3).

Among patients with CCI, the median ICU length of stay before CCI onset was 6 days (IQR 0–17). This interval reflects the time from admission to our center and should not be interpreted as the total duration of critical illness, as most patients were transferred after prior treatment in other hospitals. The median duration of CCI was 21.0 days (8.0–36.0). Patients experienced a median of 1 ICS episode (1,2) (maximum 12), with a total median ICS duration of 4 days (2–8) (maximum 146 days). The median proportion of time spent with ICS was 15% (7–32%) of the total ICU stay and 29% (11–57%) of the CCI period. Swimmer plots illustrating ICU time courses are shown in Figure 2 and Figures S1–S17.

### 3.2. Mortality Frequency and Mortality Density Analysis

Mortality frequency was assessed in patients without CCI, in the overall CCI group, and separately during the non-critical and critical periods of CCI. Patients with CCI had a significantly higher mortality rate than those without CCI (29.63% vs. 5.83%; RR 5.08, 95% CI 4.21–6.13; *p* < 0.001). Mortality was also significantly higher during both the non-critical and critical CCI periods compared with the no-CCI group. No significant difference in mortality was observed between the critical and non-critical CCI periods (RR 1.21, 95% CI 0.96–1.53; *p* = 0.112) (Table 4).

Patients with CCI had a higher mortality density than those without CCI (10.21 vs. 2.50 per 1000 patient-days; IRR 4.09, 95% CI 3.33–5.01; *p* < 0.001). Of the total 23,796 patient-days of CCI, 22.15% (5271) occurred during critical episodes and 77.85% (18,525) during non-critical episodes. Mortality density was higher during both the non-critical and critical CCI periods compared with patients without CCI, with the highest value observed during the critical CCI period (25.23 per 1000 patient-days). Mortality density was also higher in the critical than in the non-critical CCI period (IRR 4.25, 95% CI 3.30–5.47; *p* < 0.001) (Table 5, Figure 3).

## 4. Discussion

### 4.1. Key Findings

The objective of this study was to compare mortality among patients without CCI, patients with CCI overall, and CCI patients during critical and non-critical periods. Analysis of 3374 patients revealed a CCI prevalence of 24.3%. Patients with CCI were older (68 vs. 60 years, *p* < 0.001) and had higher illness severity at admission (lower GCS and FOUR scores, higher SOFA-2 score, all *p* < 0.001). ICU mortality was significantly higher in patients with CCI (29.6% vs. 5.8%, *p* < 0.001; OR 6.80, 95% CI 5.44–8.50), as were rates of nosocomial pneumonia (72.6% vs. 47.7%), sepsis (28.7% vs. 17.6%), multiple organ failure (80.5% vs. 44.9%), and the need for mechanical ventilation (79.5% vs. 48.2%) and vasopressor support (41.1% vs. 12.3%) (all *p* < 0.001). The CCI group also had a longer ICU stay (30 vs. 19 days, *p* < 0.001). When comparing CCI periods, critical episodes accounted for 22.15% (5271/23,796) of CCI patient-days, non-critical for 77.85% (18,525/23,796). Mortality incidence did not differ significantly between critical and non-critical periods (16.22% vs. 13.41%, RR 1.21, 95% CI 0.96–1.53, *p* = 0.112). However, mortality density was more than four times higher during the critical period than during the non-critical period (25.23 vs. 5.94 per 1000 patient-days; IRR 4.25, 95% CI 3.30–5.47, *p* < 0.001) and ten times higher compared with the no-CCI group (IRR 10.10, 95% CI 8.00–12.77, *p* < 0.001).

### 4.2. Relationship with Previous Studies

The ICU mortality observed in our CCI cohort (29.6%) aligns closely with previously reported data. Dong Hu and colleagues found a mortality of 28.3% among patients with the ICS triad [13], and Na Yang et al. reported 24.2% [14]. In the study by Peizhao Liu et al., ICU mortality was 18.3% and in-hospital mortality 23.3%—somewhat lower yet still comparable [15]. Both median ICU stay (20.6 vs. 30 days) and total hospital stay (27 vs. 39 days) were shorter in that study than in our cohort [15].

A lower mortality rate (13.9%) was reported by Nakamura and colleagues, a difference likely attributable to their inclusion criterion of an ICU stay exceeding 14 days, whereas our analysis included all patients who survived the first 24 h of admission [16].

Notably, the prevalence of CCI varies substantially across cohorts. Demirkiran et al. documented a CCI prevalence of 10.7% (compared with our 24.3%) and a sepsis rate of 25.4% (similar to our 28.7%). However, their ICU mortality reached 61.2%, which is more than double our rate [17].

In contrast, Ballesteros and colleagues reported a CCI prevalence of 14%, a vasopressor requirement of 42.6% (close to our 41.1%), and a median ICU stay of 29.8 days (virtually identical to our 30 days). Yet their ICU mortality was only 8.47%, substantially lower than ours. This marked difference likely stems from the underlying diagnosis (polytrauma in their cohort versus predominantly neurological conditions in ours) and possibly lower severity of illness at admission [18].

Taken together, these comparisons underscore the wide variability in CCI outcomes across studies, ranging from less than 10% to over 60%, a finding consistent with the large meta-analysis by Ohbe and colleagues, who attributed this heterogeneity largely to the absence of universally accepted diagnostic criteria for chronic critical illness [5].

### 4.3. Significance of Study Findings

The significance of the obtained results is determined by several conclusions.

#### 4.3.1. The Development of Chronic Critical Illness and Its Temporal Course Affect Mortality Risk

In this study, the development of CCI was associated with a five-fold increase in mortality (29.6% vs. 5.8%) and a four-fold increase in mortality density (10.21 vs. 2.50 per 1000 patient-days). Our findings indicate that the course of chronic critical illness is temporally heterogeneous, with alternating critical and non-critical periods. Notably, non-critical episodes occupy substantially more of the CCI time course, accounting for roughly four-fifths of all CCI patient-days, while critical episodes account for only one-fifth. Nevertheless, critical episodes are associated with a four-fold higher mortality density compared with non-critical episodes. Thus, we observe a divergence in mortality risk during CCI, which supports the concept of critical episodes. These findings suggest that deaths cluster within short periods of simultaneous decompensation of all three components of CCI pathophysiology (inflammation, catabolism, and immunosuppression). In contrast, non-critical episodes, which reflect partial compensation, represent a more stable clinical state. From a clinical perspective, these findings suggest that serial assessment of routinely available CRP, albumin, and lymphocyte count may help characterize changes in the biological state of patients with CCI and identify periods associated with increased mortality density. However, the present observational study does not establish a clinical monitoring algorithm or treatment threshold, and prospective external validation is required before this approach can be translated into routine clinical practice.

#### 4.3.2. Defining Diagnostic Criteria for CCI

These findings raise an important question: should CCI onset be defined as the time when the ICS triad is first detected? This question should be considered only after the acute phase of the underlying disease has resolved, since the same laboratory abnormalities observed during acute severe illness may primarily reflect the intensity of the systemic response and overall disease severity rather than the development of chronic critical illness. Thus, we do not suggest that the presence of the ICS triad alone is sufficient to define CCI irrespective of the phase of illness.

Once this distinction is made, a second question arises regarding the subsequent course of CCI: does it represent a continuously persistent condition, or rather a state characterized by recurrent episodes of simultaneous inflammation, catabolism, and immunosuppression? Patients may experience intermittent triad episodes separated by more stable intervals in which one or more components are no longer present. In this sense, CCI may not represent a uniformly continuous state, but rather a dynamic condition with alternating periods of biological decompensation and partial compensation.

The answer becomes clearer when we examine the non-critical periods. Even when the triad is not fully present, these intervals are associated with a two-fold higher mortality frequency and mortality density compared with patients who never experience a triad episode (those without CCI). This indicates that the first episode of decompensation, which creates a “vicious circle” of inflammation, catabolism, and immunosuppression, fundamentally alters the patient’s physiological state. Even during more stable intervals, survival prospects remain dramatically reduced. Therefore, our findings support considering the first detection of the ICS triad after resolution of the acute phase of the underlying disease as a potential biological marker of CCI onset. However, this finding requires confirmation in further clinical trials.

### 4.4. Strengths and Limitations

This study has several important strengths. To our knowledge, it is the first to directly compare mortality frequency and mortality density between critical and non-critical periods of chronic critical illness, offering a dynamic view of CCI course rather than a static snapshot. The use of mortality density (deaths per 1000 patient-days) alongside conventional mortality frequency provides a more nuanced understanding of risk, particularly given the substantial difference in time spent in critical versus non-critical episodes. The large sample size (3374 patients, including 820 with CCI) and the detailed clinical and laboratory characterization at ICU admission allowed robust comparisons between groups. We applied a strict Bonferroni correction to reduce the risk of type I error for the multiple pairwise comparisons in the mortality analyses. The swimmer plot visualization transparently illustrates the temporal heterogeneity of ICS episodes, supporting the concept of alternating critical and non-critical phases. Notably, the use of routine laboratory parameters (CRP, albumin, lymphocyte count) is a key strength, as these tests are universally available, inexpensive, and part of standard clinical care, which enhances the real-world applicability of our diagnostic criteria for CCI onset.

Several limitations should also be acknowledged. The single-center, retrospective design limits the generalizability of our findings to other ICU populations with different case mixes or treatment protocols. Additionally, the reported comparisons were unadjusted and therefore do not account for potential confounding by age, baseline disease severity, comorbidities, or changes in clinical status during the ICU course. No multivariable, time-to-event, or other longitudinal model was performed to estimate an independent association between CCI state and mortality. Accordingly, the observed differences should be interpreted as associations rather than independent effects of the ICS-defined state. Furthermore, CRP, albumin, and absolute lymphocyte count were used as predefined components of the operational ICS triad and were not evaluated as independent prognostic or diagnostic markers. Their individual temporal trajectories and independent contributions to outcome were beyond the scope of the present analysis. Moreover, the frequency and timing of laboratory measurements were determined by clinical necessity rather than a fixed research schedule, which could introduce variability in the detection of ICS episodes; however, this reflects real-world practice and does not undermine the main conclusions. The available RICD data are limited to the index hospitalization and do not include systematic post-discharge follow-up. Consequently, 28-day, 90-day, and post-hospital mortality could not be evaluated, and ICU mortality may provide only a partial representation of the long-term burden of CCI. The present study also did not directly compare the proposed ICS-based approach with conventional time-based definitions of CCI. Therefore, our findings cannot establish whether the ICS-based approach is superior to or can replace existing temporal criteria. Direct comparison of alternative CCI definitions using prespecified criteria and appropriately adjusted analyses should be addressed in future studies. Finally, the results should be validated externally using an independent patient cohort, preferably from a prospective multicenter study.

### 4.5. Future Prospects

Our findings point to several directions for future research. First, future studies should include longitudinal follow-up of patients discharged alive to evaluate long-term survival and other clinically relevant post-hospital outcomes. Such data would provide a more comprehensive assessment of the clinical course and consequences of CCI beyond the ICU stay.

Second, it remains unclear whether a critical episode of CCI acts as a direct trigger of clinical deterioration or merely reflects the laboratory manifestation of an underlying complication. To address this question, the incidence of hospital-acquired complications, including nosocomial pneumonia and sepsis, should be compared between critical and non-critical periods.

Third, future research should investigate strategies to prevent the occurrence of critical episodes or reduce their duration. Given that critical episodes account for a disproportionately high mortality density, even a modest reduction in their frequency or length could translate into meaningful improvements in patient survival. Studies are needed to determine whether close monitoring of laboratory parameters (such as CRP, albumin, and lymphocyte count) can predict the impending onset of a critical episode, allowing preemptive interventions. Future studies should also evaluate the longitudinal trajectories and independent prognostic contribution of individual ICS components using appropriately adjusted time-dependent models. Furthermore, randomized trials could evaluate whether optimized nutritional support, infection prevention measures, or immunomodulatory therapies administered during non-critical periods can lower the risk of transitioning into a critical episode.

## 5. Conclusions

Patients with CCI have a fivefold higher mortality (29.6% vs. 5.8%) than those without CCI. The course of CCI consists of alternating critical and non-critical episodes. Critical episodes account for only 22.15% of CCI patient days yet carry a fourfold higher mortality density than non-critical periods and a tenfold higher mortality density than the no CCI group (25.23 vs. 2.50 per 1000 patient days). Even non critical periods are associated with a twofold higher mortality compared with no CCI. These findings support defining CCI onset as the first detection of the ICS triad.

## Figures and Tables

**Figure 1:**
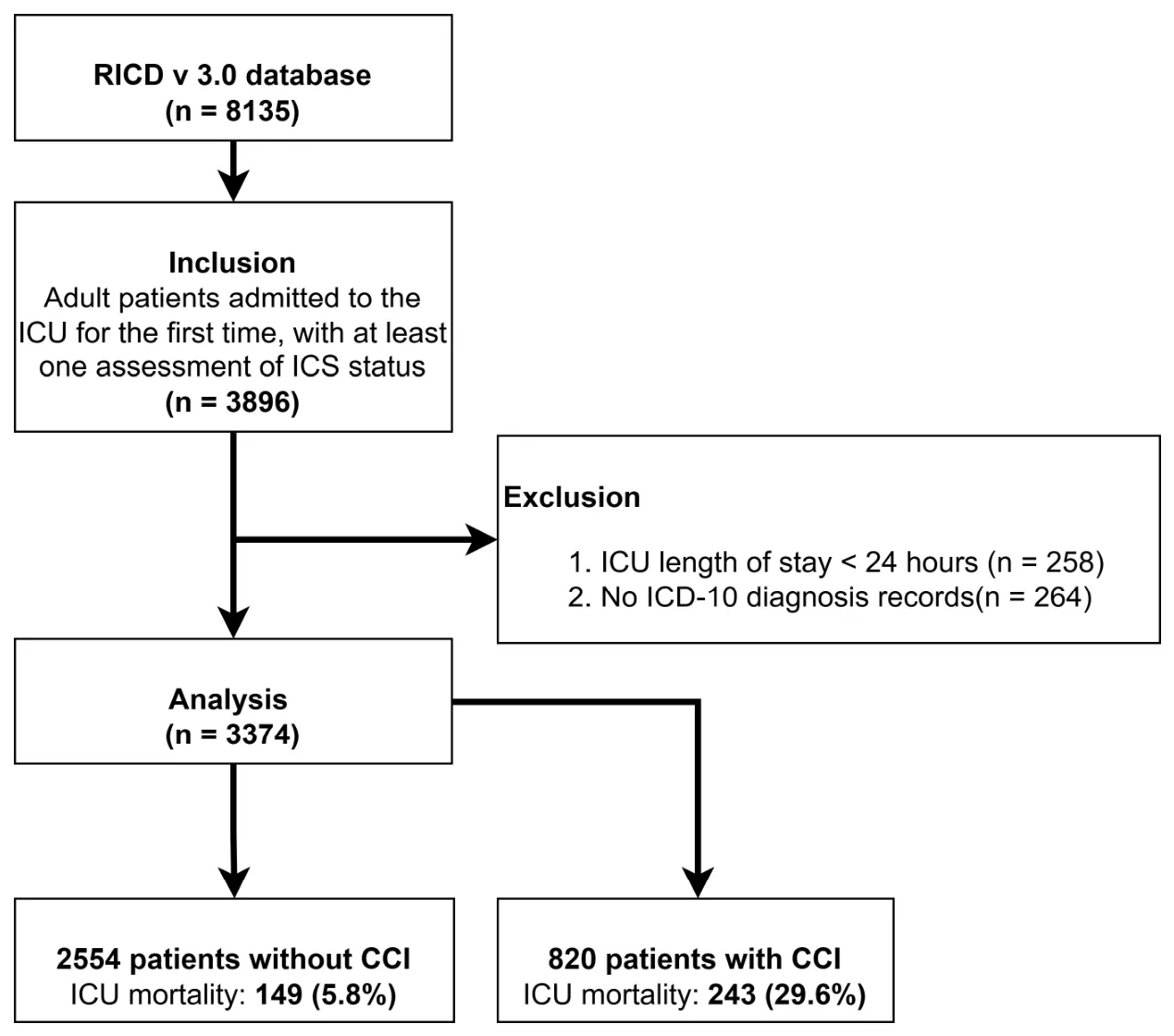
Study flow diagram.

**Figure 2:**
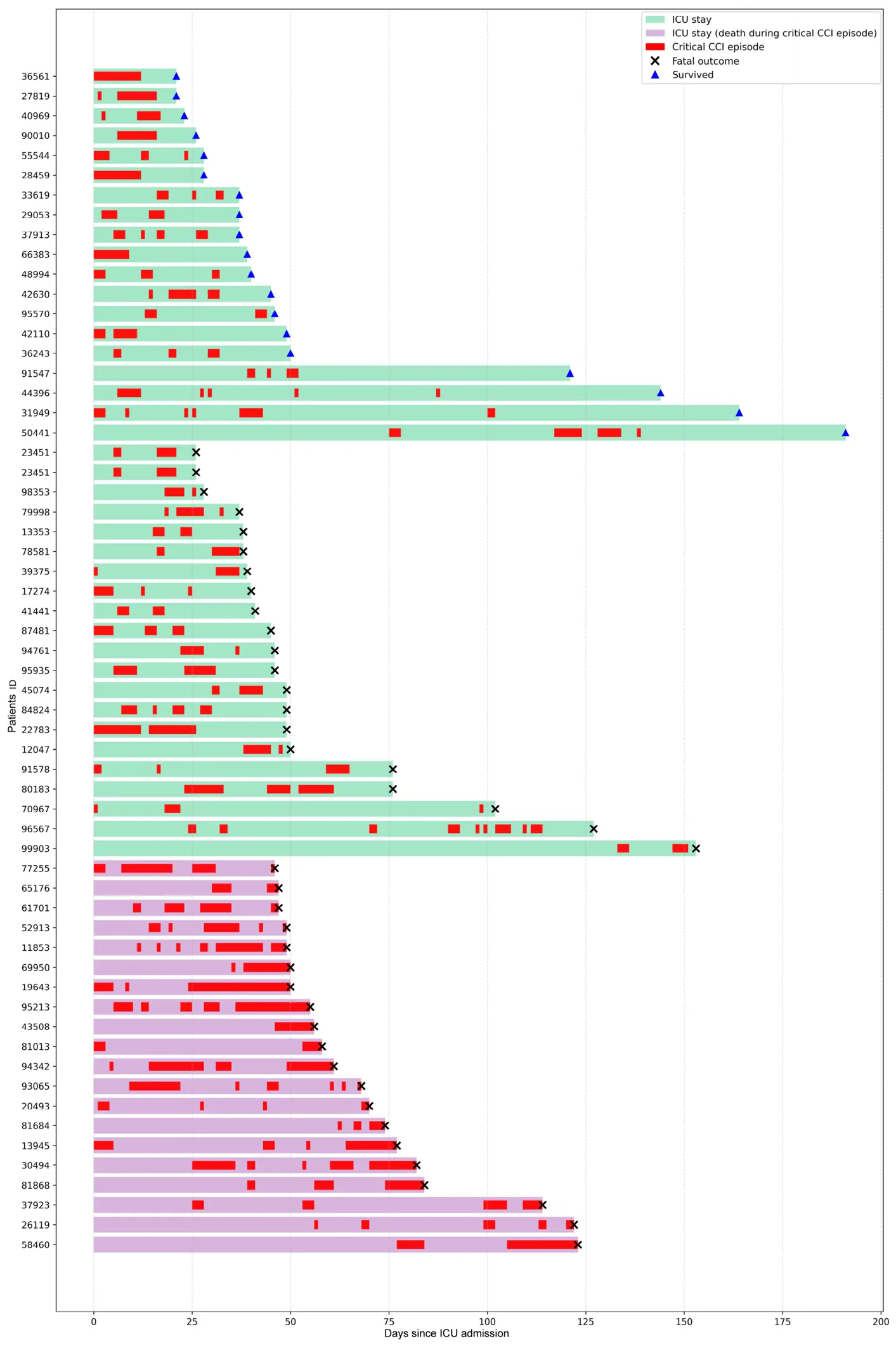
Swimmer plots of ICU stay and critical CCI episodes.

**Figure 3:**
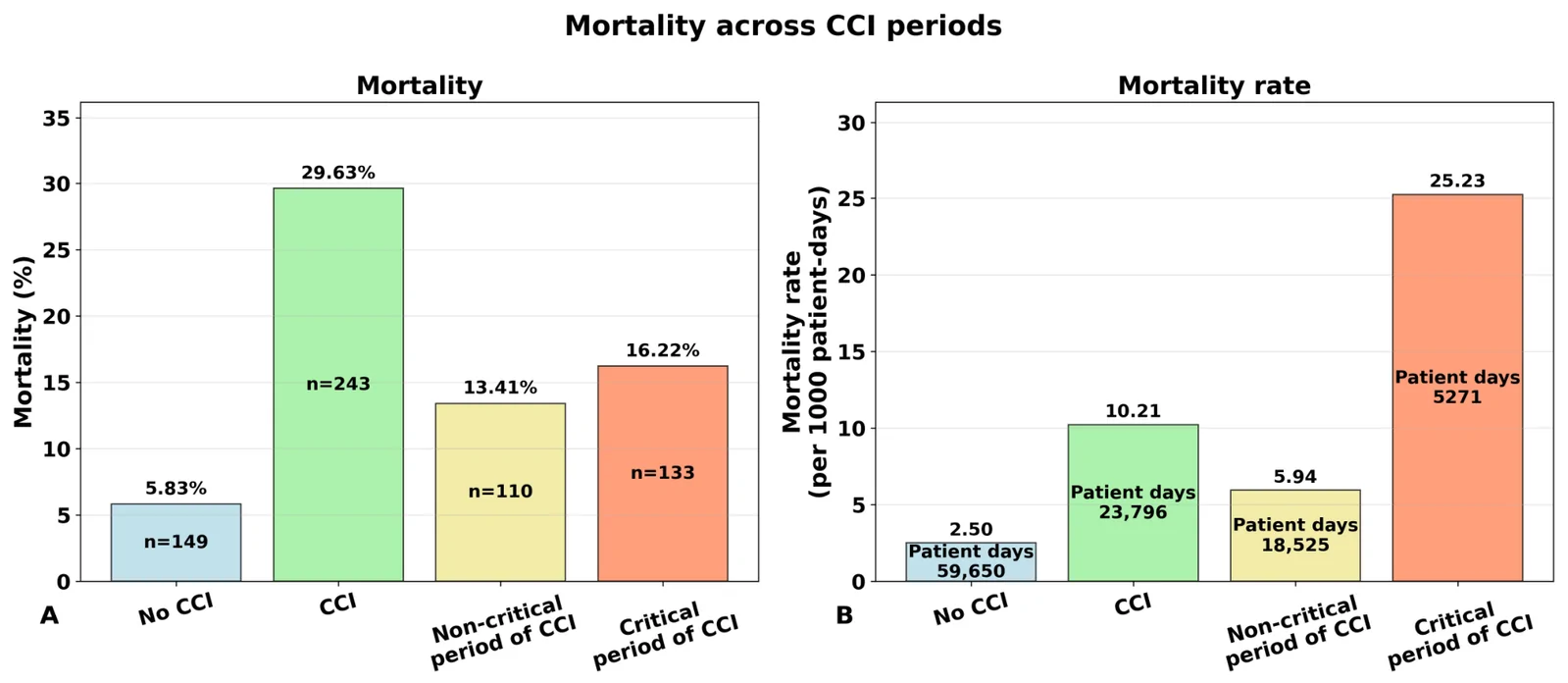
Mortality and mortality rate in chronic critical illness.

**Table 1:** Baseline characteristics of patients with and without CCI.

Parameter	No CCI (N = 2554)	CCI (N = 820)	*p*-Value	OR (95% CI)
Clinical and demographic data				
Sex (male), n (%)	1470 (57.6%)	434 (52.9%)	0.020	0.83 (0.71; 0.97)
Age, years	60 (45; 71)	68 (55; 77)	<0.001	
BMI, kg/m^2^	25.3 (21.9; 29.2)	25.1 (22.1; 29.3)	0.680	
State on admission				
Ischemic stroke, n (%)	1172 (45.9%)	403 (49.1%)	0.104	1.14 (0.97; 1.33)
Hemorrhagic stroke, n (%)	409 (16.0%)	153 (18.7%)	0.077	1.20 (0.98; 1.48)
Traumatic brain injury, n (%)	453 (17.7%)	114 (13.9%)	0.011	0.75 (0.60; 0.94)
Anoxic brain injury, n (%)	120 (4.7%)	59 (7.2%)	0.006	1.57 (1.14; 2.17)
Polytrauma, n (%)	123 (4.8%)	28 (3.4%)	0.091	0.70 (0.46; 1.06)
Pneumonia, n (%)	977 (38.3%)	474 (57.8%)	<0.001	2.21 (1.88; 2.59)
Anamnestic data				
Cerebrovascular disease, n (%)	101 (4.0%)	38 (4.6%)	0.394	1.18 (0.81; 1.73)
Polyneuropathy, n (%)	126 (4.9%)	39 (4.8%)	0.838	0.96 (0.67; 1.39)
Coronary artery disease, n (%)	731 (28.6%)	340 (41.5%)	<0.001	1.77 (1.50; 2.08)
Myocardial infarction, n (%)	16 (0.6%)	5 (0.6%)	0.958	0.97 (0.36; 2.66)
Atrial fibrillation, n (%)	140 (5.5%)	66 (8.0%)	0.008	1.51 (1.11; 2.05)
CHF, n (%)	384 (15.0%)	190 (23.2%)	<0.001	1.70 (1.40; 2.07)
Arterial hypertension, n (%)	1716 (67.2%)	597 (72.8%)	0.003	1.31 (1.10; 1.56)
CKD, n (%)	169 (6.6%)	77 (9.4%)	0.008	1.46 (1.10; 1.94)
COPD, n (%)	39 (1.5%)	14 (1.7%)	0.718	1.12 (0.61; 2.07)
Type 1 diabetes, n (%)	15 (0.6%)	10 (1.2%)	0.066	2.09 (0.94; 4.67)
Type 2 diabetes, n (%)	255 (10.0%)	88 (10.7%)	0.538	1.08 (0.84; 1.40)
Anemia, n (%)	263 (10.3%)	112 (13.7%)	0.008	1.38 (1.09; 1.75)
Coagulopathy, n (%)	16 (0.6%)	16 (2.0%)	<0.001	3.16 (1.57; 6.34)
Malignant tumor, n (%)	49 (1.9%)	29 (3.5%)	0.007	1.87 (1.18; 2.99)
Transferred from another hospital, n (%)	2337 (91.5%)	773 (94.3%)	0.010	1.53 (1.10; 2.12)
Scales				
GCS, points	13 (10; 15)	10 (8; 13)	<0.001	
FOUR, points	16 (13; 16)	13 (10; 15)	<0.001	
SOFA-2, points	2 (0; 3)	3 (2; 5)	<0.001	
DRS, points	18 (14; 22)	22 (19; 24)	<0.001	
CRS-R, points	18 (9; 22)	10 (5; 17)	<0.001	
mRS, points	5 (4; 5)	5 (5; 5)	<0.001	

Note: Continuous variables are presented as median (interquartile range) and categorical variables as n (%). Between-group comparisons were performed using the Mann–Whitney U test for continuous variables and Pearson’s chi-square test for categorical variables. For categorical variables, odds ratios (ORs) with 95% confidence intervals (CIs) were additionally calculated; 95% CIs were obtained on the logarithmic scale using Wald standard errors. CCI, chronic critical illness; OR, odds ratio; CI, confidence interval; BMI, body mass index; CHF, chronic heart failure; CKD, chronic kidney disease; COPD, chronic obstructive pulmonary disease; GCS, Glasgow Coma Scale; FOUR, Full Outline of UnResponsiveness; SOFA-2, Sequential Organ Failure Assessment 2; DRS, Disability Rating Scale; CRS-R, Coma Recovery Scale–Revised; mRS, Modified Rankin Scale.

**Table 2:** Laboratory parameters at ICU admission in patients with and without CCI.

Parameter	No CCI (N = 2554)	CCI (N = 820)	*p*-Value
RBC, ×10^12^/L	4.0 (3.5; 4.5)	3.6 (3.2; 4.2)	<0.001
Hemoglobin, g/L	117 (101; 133)	106 (94; 121)	<0.001
Hematocrit, %	35.5 (30.9; 40.3)	32.6 (28.8; 37.3)	<0.001
MCH, pg	29.7 (28.4; 30.9)	29.5 (28.3; 30.9)	0.275
MCHC, g/L	330 (323; 336)	326 (319; 333)	<0.001
MCV, fL	89.7 (86.5; 93.3)	90.4 (86.4; 94.2)	0.028
RDW-CV, %	14.6 (13.6; 16.0)	15.2 (14.0; 16.8)	<0.001
RDW-SD, fL	46.8 (43.7; 51.0)	49.0 (45.5; 53.8)	<0.001
Platelets, ×10^9^/L	286 (223; 369)	251 (188; 343)	<0.001
Plateletcrit, %	0.26 (0.21; 0.34)	0.24 (0.18; 0.31)	<0.001
MPV, fL	9.1 (8.2; 10.2)	9.2 (8.4; 10.4)	0.0501
PDW, fL	16.4 (13.0; 16.9)	16.5 (14.4; 17.2)	<0.001
WBC, ×10^9^/L	8.9 (6.9; 11.6)	9.6 (7.2; 12.8)	<0.001
Lymphocytes (abs.), ×10^9^/L	1.49 (1.10; 1.93)	0.90 (0.60; 1.30)	<0.001
Monocytes (abs.), ×10^9^/L	0.66 (0.50; 0.90)	0.60 (0.40; 0.80)	<0.001
Neutrophils (abs.), ×10^9^/L	6.3 (4.6; 8.9)	7.7 (5.5; 10.6)	<0.001
Basophils (abs.), ×10^9^/L	0.05 (0.01; 0.10)	0.03 (0.00; 0.10)	<0.001
Eosinophils (abs.), ×10^9^/L	0.11 (0.10; 0.24)	0.10 (0.02; 0.20)	<0.001
Lymphocytes, %	16.8 (11.6; 23.5)	9.7 (6.1; 14.5)	<0.001
Monocytes, %	7.5 (5.9; 9.2)	6.5 (4.5; 8.4)	<0.001
Neutrophils, %	72.2 (64.3; 79.1)	81.5 (74.3; 86.6)	<0.001
Basophils, %	0.50 (0.30; 0.80)	0.40 (0.20; 0.70)	<0.001
Eosinophils, %	1.45 (0.60; 2.80)	0.80 (0.20; 2.10)	<0.001
ESR, mm/h	34 (20; 48)	45 (31; 55)	<0.001
Total protein, g/L	62.8 (57.7; 67.7)	57.5 (52.1; 62.9)	<0.001
Albumin, g/L	32.8 (28.6; 36.6)	28.2 (24.9; 31.8)	<0.001
C-reactive protein, mg/L	33.3 (11.1; 69.3)	69.5 (36.8; 132.8)	<0.001
Total bilirubin, µmol/L	10.8 (8.1; 14.6)	12.2 (9.1; 17.0)	<0.001
Direct bilirubin, µmol/L	2.2 (1.5; 3.4)	2.9 (1.9; 4.8)	<0.001
AST, U/L	29.4 (20.5; 44.5)	32.1 (21.6; 48.5)	<0.001
ALT, U/L	28.1 (16.9; 49.5)	27.9 (16.6; 49.2)	0.799
ALP, U/L	86.6 (66.8; 118.3)	88.9 (68.5; 123.4)	0.149
α-Amylase, U/L	52.7 (36.9; 75.6)	50.5 (34.2; 81.9)	0.470
Creatinine, µmol/L	76.6 (61.6; 96.3)	77.8 (58.9; 111.5)	0.183
Urea, mmol/L	5.7 (3.9; 8.7)	7.4 (4.7; 12.6)	<0.001
Glucose, mmol/L	5.6 (5.1; 6.5)	6.0 (5.2; 7.6)	<0.001
Potassium, mmol/L	4.0 (3.6; 4.3)	3.9 (3.4; 4.2)	<0.001
Sodium, mmol/L	138 (135; 140)	138 (135; 145)	<0.001
Calcium, mmol/L	2.2 (2.0; 2.3)	2.0 (1.9; 2.2)	<0.001
Iron, µmol/L	5.9 (3.9; 8.5)	4.7 (3.2; 7.3)	<0.001
aPTT, s	27.4 (24.3; 31.5)	28.2 (24.6; 32.4)	0.012
INR	1.1 (1.1; 1.3)	1.2 (1.1; 1.4)	<0.001
Thrombin time, s	17.2 (14.3; 19.2)	17.3 (14.9; 19.4)	0.102
Prothrombin time, s	12.1 (11.3; 13.4)	12.8 (11.6; 14.4)	<0.001
Prothrombin ratio	1.2 (1.1; 1.3)	1.3 (1.1; 1.4)	<0.001
Prothrombin activity (by Quick), %	78.0 (63.9; 94.3)	73.1 (57.8; 89.2)	<0.001
Fibrinogen (Clauss method), g/L	5.0 (3.9; 6.5)	5.3 (4.0; 7.0)	0.002

Note: Continuous variables are presented as median (interquartile range). Between-group comparisons were performed using the Mann–Whitney U test. MCH, mean corpuscular hemoglobin; MCHC, mean corpuscular hemoglobin concentration; MCV, mean corpuscular volume; RBC, red blood cells; RDW-CV, red cell distribution width–coefficient of variation; RDW-SD, red cell distribution width–standard deviation; MPV, mean platelet volume; PDW, platelet distribution width; ESR, erythrocyte sedimentation rate; AST, aspartate aminotransferase; ALT, alanine aminotransferase; ALP, alkaline phosphatase; aPTT, activated partial thromboplastin time; INR, international normalized ratio; WBC, white blood cells.

**Table 3:** ICU outcomes and hospital course characteristics.

Parameter	No CCI (N = 2554)	CCI (N = 820)	*p*-Value	OR (95% CI)
ICU mortality, n (%)	149 (5.8%)	243 (29.6%)	<0.001	6.80 (5.44; 8.50)
Nosocomial pneumonia, n (%)	1218 (47.7%)	595 (72.6%)	<0.001	2.90 (2.44; 3.44)
Sepsis, n (%)	449 (17.6%)	235 (28.7%)	<0.001	1.88 (1.57; 2.26)
Multiple organ failure, n (%)	1148 (44.9%)	660 (80.5%)	<0.001	5.05 (4.18; 6.11)
Need for mechanical ventilation, n (%)	1230 (48.2%)	652 (79.5%)	<0.001	4.18 (3.47; 5.03)
Need for vasopressor support, n (%)	314 (12.3%)	337 (41.1%)	<0.001	4.98 (4.15; 5.97)
Duration of ICU stay, days	19 (7; 29)	30 (21; 55)	<0.001	
Duration of hospitalization, days	37 (22; 58)	39 (22; 67)	0.147	

Note: Continuous variables are presented as median (interquartile range) and categorical variables as n (%). Between-group comparisons were performed using the Mann–Whitney U test for continuous variables and Pearson’s chi-square test for categorical variables. For categorical variables, odds ratios (ORs) with 95% confidence intervals (CIs) were additionally calculated; 95% CIs were obtained on the logarithmic scale using Wald standard errors. CCI, chronic critical illness; OR, odds ratio; CI, confidence interval; ICU, intensive care unit.

**Table 4:** Mortality frequency in patients with and without CCI and across CCI periods.

Group No.	Group Description	Mortality Frequency	Mortality Proportion, %
1	No CCI	149/2554	5.83
2	All patients with CCI	243/820	29.63
3	CCI, non-critical period	110/820	13.41
4	CCI, critical period	133/820	16.22
Comparison			
Comparison pair	RR	95% CI	*p*-value
2/1	5.08	4.21–6.13	<0.001
3/1	2.30	1.82–2.90	<0.001
4/1	2.78	2.23–3.46	<0.001
4/3	1.21	0.96–1.53	0.112

Note: Relative risks (RRs) were calculated as ratios of mortality proportions. The corresponding 95% confidence intervals were calculated on the logarithmic scale using Wald standard errors, and *p*-values were obtained using two-sided Wald tests of RR = 1. CCI, chronic critical illness; RR, relative risk; CI, confidence interval; comparisons involving the overall CCI group and CCI subperiods were interpreted as exploratory because these categories were not fully independent; Bonferroni-adjusted significance threshold: *p* < 0.0125.

**Table 5:** Mortality density in patients with and without CCI and across CCI periods.

Group No.	Group Description	Patient-Days	Mortality Density per 1000 Patient-Days
1	No CCI	59,650	2.5
2	All patients with CCI	23,796	10.21
3	CCI, non-critical period	18,525	5.94
4	CCI, critical period	5271	25.23
Comparison			
Comparison pair	IRR	95% CI	*p*-value
2/1	4.09	3.33–5.01	<0.001
3/1	2.38	1.86–3.04	<0.001
4/1	10.10	8.00–12.77	<0.001
3/2	0.58	0.46–0.73	<0.001
4/2	2.47	2.00–3.05	<0.001
4/3	4.25	3.30–5.47	<0.001

Note: Mortality density was calculated as the number of ICU deaths per 1000 patient-days. Incidence rate ratios (IRRs) were calculated as ratios of mortality rates. The corresponding 95% confidence intervals were calculated on the logarithmic scale using Wald standard errors, and *p*-values were obtained using two-sided Wald tests of IRR = 1. CCI, chronic critical illness; IRR, incidence rate ratio; CI, confidence interval; comparisons involving the overall CCI group and CCI subperiods were interpreted as exploratory because these categories were not fully independent; Bonferroni-adjusted significance threshold: *p* < 0.0083.

## Data Availability

Publicly and partially available datasets were analyzed in this study. The RICD dataset can be obtained upon request at https://fnkcrr-database.ru/.

## Supplementary Materials

The following supporting information can be downloaded at: https://www.mdpi.com/article/10.3390/jcm15207864/s1, Table S1: STROBE Statement—Checklist; Figure S1: Swimmer plots of ICU stay and critical CCI episodes (part 1); Figure S2: Swimmer plots of ICU stay and critical CCI episodes (part 2); Figure S3: Swimmer plots of ICU stay and critical CCI episodes (part 3); Figure S4: Swimmer plots of ICU stay and critical CCI episodes (part 4); Figure S5: Swimmer plots of ICU stay and critical CCI episodes (part 5); Figure S6: Swimmer plots of ICU stay and critical CCI episodes (part 6); Figure S7: Swimmer plots of ICU stay and critical CCI episodes (part 7); Figure S8: Swimmer plots of ICU stay and critical CCI episodes (part 8); Figure S9: Swimmer plots of ICU stay and critical CCI episodes (part 9); Figure S10: Swimmer plots of ICU stay and critical CCI episodes (part 10); Figure S11: Swimmer plots of ICU stay and critical CCI episodes (part 11); Figure S12: Swimmer plots of ICU stay and critical CCI episodes (part 12); Figure S13: Swimmer plots of ICU stay and critical CCI episodes (part 13); Figure S14: Swimmer plots of ICU stay and critical CCI episodes (part 14); Figure S15: Swimmer plots of ICU stay and critical CCI episodes (part 15); Figure S16: Swimmer plots of ICU stay and critical CCI episodes (part 16); Figure S17: Swimmer plots of ICU stay and critical CCI episodes (part 17).

## Abbreviations

The following abbreviations are used in this manuscript:

ALP	Alkaline phosphatase
ALT	Alanine aminotransferase
aPTT	Activated partial thromboplastin time
AST	Aspartate aminotransferase
BMI	Body mass index
CCI	Chronic critical illness
CHF	Chronic heart failure
CI	Confidence interval
CKD	Chronic kidney disease
COPD	Chronic obstructive pulmonary disease
CRS-R	Coma Recovery Scale–Revised
CRP	C-reactive protein
DRS	Disability Rating Scale
ESR	Erythrocyte sedimentation rate
FOUR	Full Outline of UnResponsiveness
FRCC ICMR	Federal Research and Clinical Center of Intensive Care Medicine and Rehabilitology
GCS	Glasgow Coma Scale
ICD-10	International Classification of Diseases, 10th Revision
ICS	Inflammation, catabolism, and immunosuppression triad
ICU	Intensive care unit
INR	International normalized ratio
IQR	Interquartile range
IRR	Incidence rate ratio
MCH	Mean corpuscular hemoglobin
MCHC	Mean corpuscular hemoglobin concentration
MCV	Mean corpuscular volume
MPV	Mean platelet volume
mRS	Modified Rankin Scale
OR	Odds ratio
PCI	Prolonged critical illness
PDW	Platelet distribution width
PICS	Persistent inflammation, immunosuppression, and catabolism syndrome
RDW-CV	Red cell distribution width–coefficient of variation
RDW-SD	Red cell distribution width–standard deviation
RICD	Russian Intensive Care Dataset
RR	Relative risk
SOFA-2	Sequential Organ Failure Assessment 2
STROBE	Strengthening the Reporting of Observational Studies in Epidemiology
