# Supplementary material for: Critical Episodes of Inflammation, Catabolism, and Immunosuppression Define Mortality Risk in Chronic Critical Illness
Source: J Clin Med. 2026 Oct 12;15(20):7864. doi: 10.3390/jcm15207864 (PMC13705316; doi:10.3390/jcm15207864)

Supplementary Information

Critical Episodes of Inflammation, Catabolism, and Immunosuppression Define Mortality Risk in Chronic Critical Illness

Valery V. Likhvantsev, Levan B. Berikashvili, Petr A. Polyakov, Mikhail Ya. Yadgarov, Alexey A. Yakovlev, Andrey V. Grechko

Federal Research and Clinical Center of Intensive Care Medicine and Rehabilitology, Moscow, Russia

Contents

Supplemental Table S1. STROBE Statement—Checklist. 3
Supplemental figure S1. Swimmer plots of ICU stay and critical CCI episodes (part 1). 6
Supplemental figure S2. Swimmer plots of ICU stay and critical CCI episodes (part 2). 7
Supplemental figure S3. Swimmer plots of ICU stay and critical CCI episodes (part 3). 8
Supplemental figure S4. Swimmer plots of ICU stay and critical CCI episodes (part 4). 9
Supplemental figure S5. Swimmer plots of ICU stay and critical CCI episodes (part 5). 10
Supplemental figure S6. Swimmer plots of ICU stay and critical CCI episodes (part 6). 11
Supplemental figure S7. Swimmer plots of ICU stay and critical CCI episodes (part 7). 12
Supplemental figure S8. Swimmer plots of ICU stay and critical CCI episodes (part 8). 13
Supplemental figure S9. Swimmer plots of ICU stay and critical CCI episodes (part 9). 14
Supplemental figure S10. Swimmer plots of ICU stay and critical CCI episodes (part 10). 15
Supplemental figure S11. Swimmer plots of ICU stay and critical CCI episodes (part 11). 16
Supplemental figure S12. Swimmer plots of ICU stay and critical CCI episodes (part 12). 17
Supplemental figure S13. Swimmer plots of ICU stay and critical CCI episodes (part 13). 18
Supplemental figure S14. Swimmer plots of ICU stay and critical CCI episodes (part 14). 19
Supplemental figure S15. Swimmer plots of ICU stay and critical CCI episodes (part 15). 20
Supplemental figure S16. Swimmer plots of ICU stay and critical CCI episodes (part 16). 21
Supplemental figure S17. Swimmer plots of ICU stay and critical CCI episodes (part 17). 22

Supplemental Table S1. STROBE Statement—Checklist.

	Item No	Recommendation	Page No. (Section(s))
**Title and abstract**	1	(*a*) Indicate the study's design with a commonly used term in the title or the abstract	Abstract — Methods
		(*b*) Provide in the abstract an informative and balanced summary of what was done and what was found	Abstract — Background/Objectives; Methods; Results; Conclusions
**Introduction**			
Background/rationale	2	Explain the scientific background and rationale for the investigation being reported	1. Introduction
Objectives	3	State specific objectives, including any prespecified hypotheses	1. Introduction
**Methods**			
Study design	4	Present key elements of study design early in the paper	2.2. Study design and setting
Setting	5	Describe the setting, locations, and relevant dates, including periods of recruitment, exposure, follow-up, and data collection	2.1. Source of data; 2.2. Study design and setting
Participants	6	(*a*) Give the eligibility criteria, and the sources and methods of selection of participants. Describe methods of follow-up	2.1. Source of data; 2.2. Study design and setting; 2.3. Data management
		(*b*) For matched studies, give matching criteria and number of exposed and unexposed	Not applicable — this was not a matched study
Variables	7	Clearly define all outcomes, exposures, predictors, potential confounders, and effect modifiers. Give diagnostic criteria, if applicable	2.2. Study design and setting; 2.3. Data management; 2.4. Statistical analysis
Data sources/ measurement	8*	For each variable of interest, give sources of data and details of methods of assessment (measurement). Describe comparability of assessment methods if there is more than one group	2.1. Source of data; 2.3. Data management
Bias	9	Describe any efforts to address potential sources of bias	2.2. Study design and setting; 2.4. Statistical analysis; 4.4. Strengths and limitations
Study size	10	Explain how the study size was arrived at	2.2. Study design and setting
Quantitative variables	11	Explain how quantitative variables were handled in the analyses. If applicable,	2.3. Data management; 2.4. Statistical analysis

		describe which groupings were chosen and why	
Statistical methods	12	(*a*) Describe all statistical methods, including those used to control for confounding	2.4. Statistical analysis
		(*b*) Describe any methods used to examine subgroups and interactions	Not applicable — no formal subgroup or interaction analyses were performed
		(*c*) Explain how missing data were addressed	2.3. Data management
		(*d*) If applicable, explain how loss to follow-up was addressed	Not applicable — retrospective in-hospital study with no post-discharge follow-up
		(*e*) Describe any sensitivity analyses	Not applicable — no sensitivity analyses were performed
**Results**			
Participants	13*	(a) Report numbers of individuals at each stage of study—e.g. numbers potentially eligible, examined for eligibility, confirmed eligible, included in the study, completing follow-up, and analysed	3.1. Patient characteristics; Figure 1. Study flow diagram
		(b) Give reasons for non-participation at each stage	3.1. Patient characteristics; Figure 1. Study flow diagram
		(c) Consider use of a flow diagram	Figure 1. Study flow diagram
Descriptive data	14*	(a) Give characteristics of study participants (e.g. demographic, clinical, social) and information on exposures and potential confounders	3.1. Patient characteristics; Table 1; Table 2; Table 3
		(b) Indicate number of participants with missing data for each variable of interest	Not reported separately; 2.3. Data management states that missing values were not imputed
		(c) Summarise follow-up time (e.g., average and total amount)	3.1. Patient characteristics; Table 3; Figure 2; Supplementary Figures 1–17
Outcome data	15*	Report numbers of outcome events or summary measures over time	3.1. Patient characteristics; 3.2. Mortality frequency and mortality density analysis; Table 3; Table 4; Table 5; Figure 3
Main results	16	(*a*) Give unadjusted estimates and, if applicable, confounder-adjusted estimates and their precision (e.g., 95% confidence interval). Make clear which confounders were adjusted for and why they were included	2.4. Statistical analysis; 3.1. Patient characteristics; 3.2. Mortality frequency and mortality density analysis; Tables 1, 3, 4, and 5
		(*b*) Report category boundaries when continuous variables were categorized	2.2. Study design and setting; 2.3. Data management

		(*c*) If relevant, consider translating estimates of relative risk into absolute risk for a meaningful time period	3.2. Mortality frequency and mortality density analysis; Table 4; Table 5
Other analyses	17	Report other analyses done—e.g. analyses of subgroups and interactions, and sensitivity analyses	Not applicable — no subgroup, interaction, or sensitivity analyses were performed
**Discussion**			
Key results	18	Summarise key results with reference to study objectives	4.1. Key findings
Limitations	19	Discuss limitations of the study, taking into account sources of potential bias or imprecision. Discuss both direction and magnitude of any potential bias	4.4. Strengths and limitations
Interpretation	20	Give a cautious overall interpretation of results considering objectives, limitations, multiplicity of analyses, results from similar studies, and other relevant evidence	4.2. Relationship with previous studies; 4.3. Significance of study findings; 4.4. Strengths and limitations; 5. Conclusions
Generalisability	21	Discuss the generalisability (external validity) of the study results	4.4. Strengths and limitations
**Other information**			
Funding	22	Give the source of funding and the role of the funders for the present study and, if applicable, for the original study on which the present article is based	Funding

*Give information separately for exposed and unexposed groups.

Note: An Explanation and Elaboration article discusses each checklist item and gives methodological background and published examples of transparent reporting. The STROBE checklist is best used in conjunction with this article (freely available on the Web sites of PLoS Medicine at http://www.plosmedicine.org/, Annals of Internal Medicine at http://www.annals.org/, and Epidemiology at http://www.epidem.com/). Information on the STROBE Initiative is available at http://www.strobe-statement.org.

Supplemental figure S1. Swimmer plots of ICU stay and critical CCI episodes (part 1).

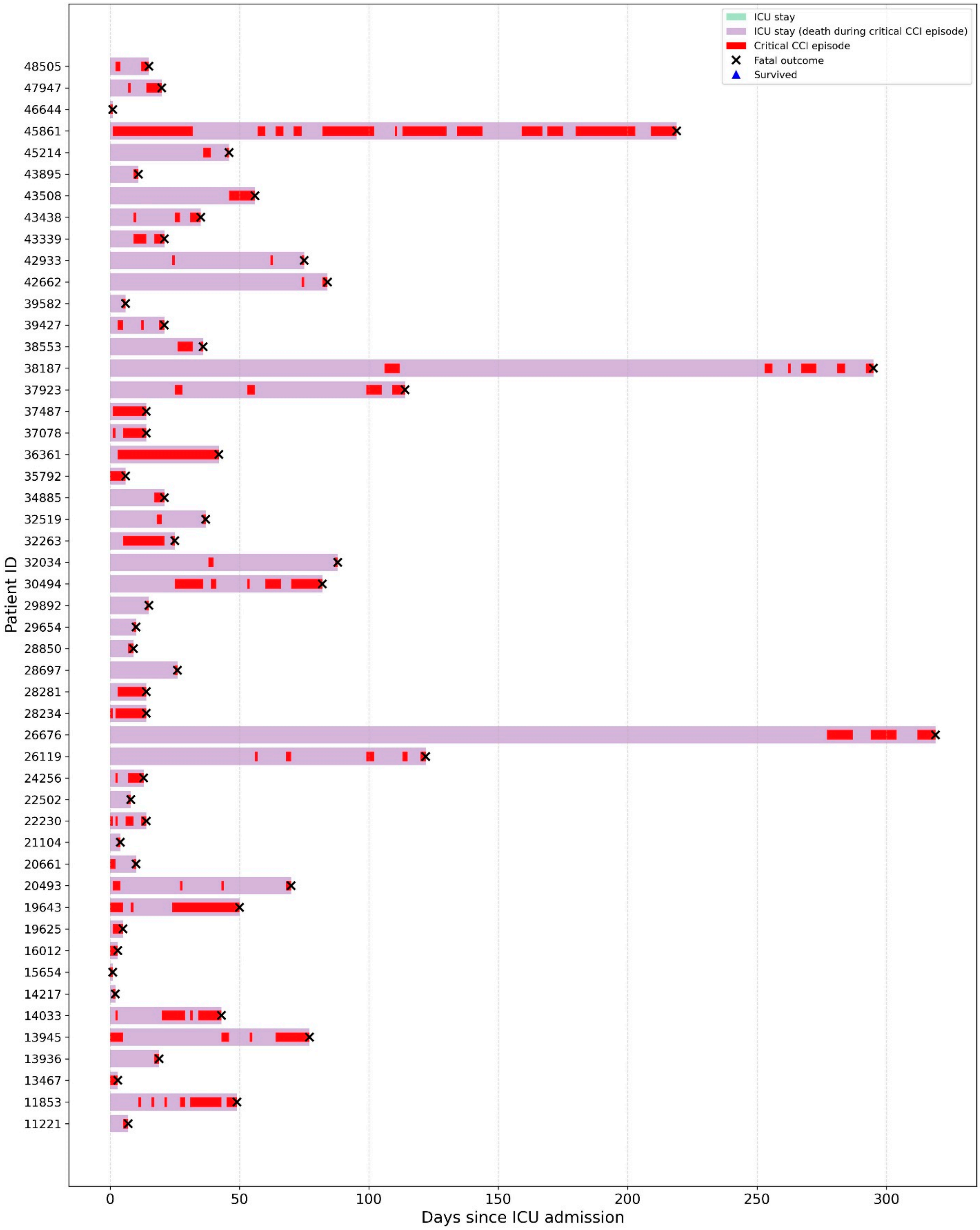

Supplemental figure S2. Swimmer plots of ICU stay and critical CCI episodes (part 2).

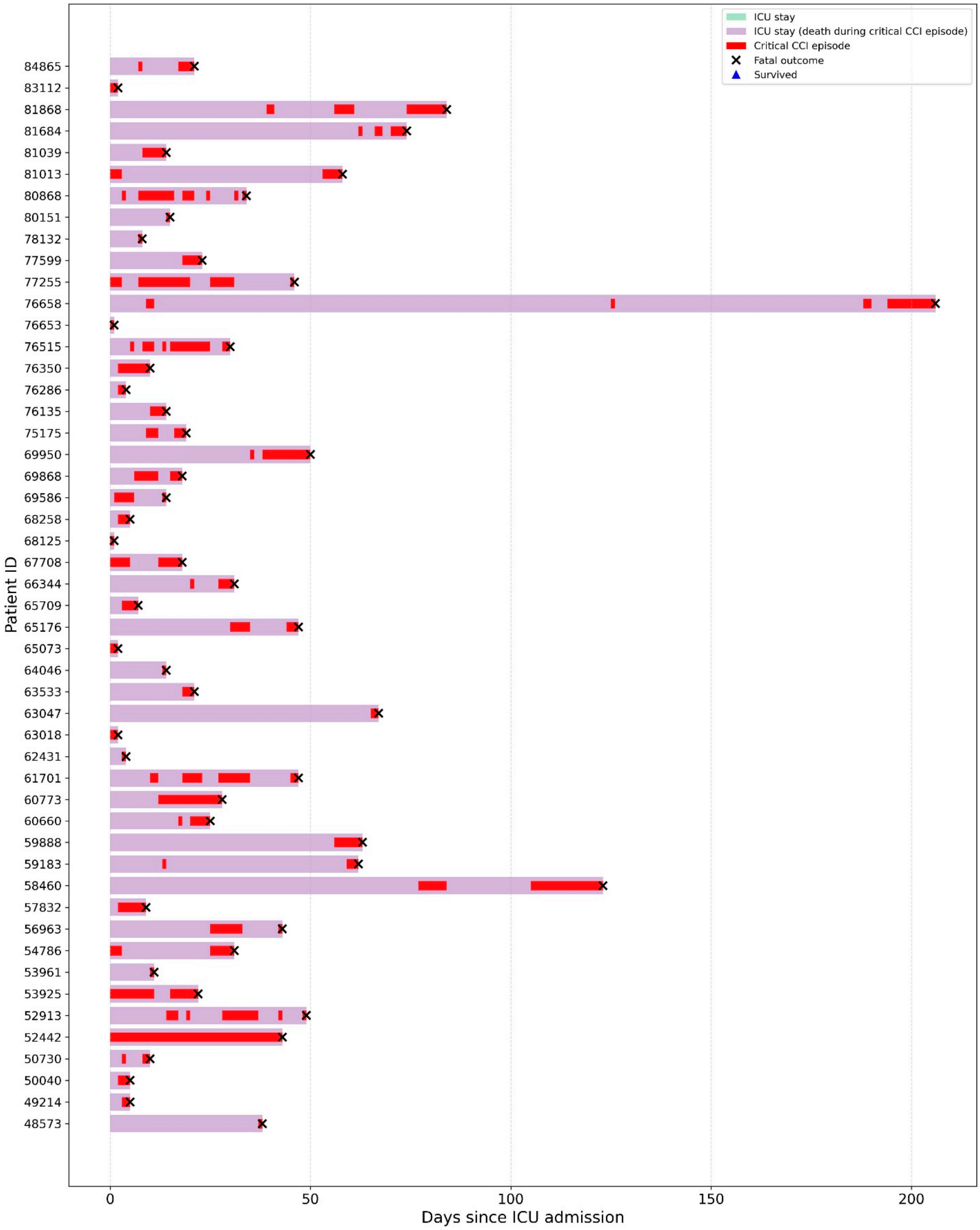

Supplemental figure S3. Swimmer plots of ICU stay and critical CCI episodes (part 3).

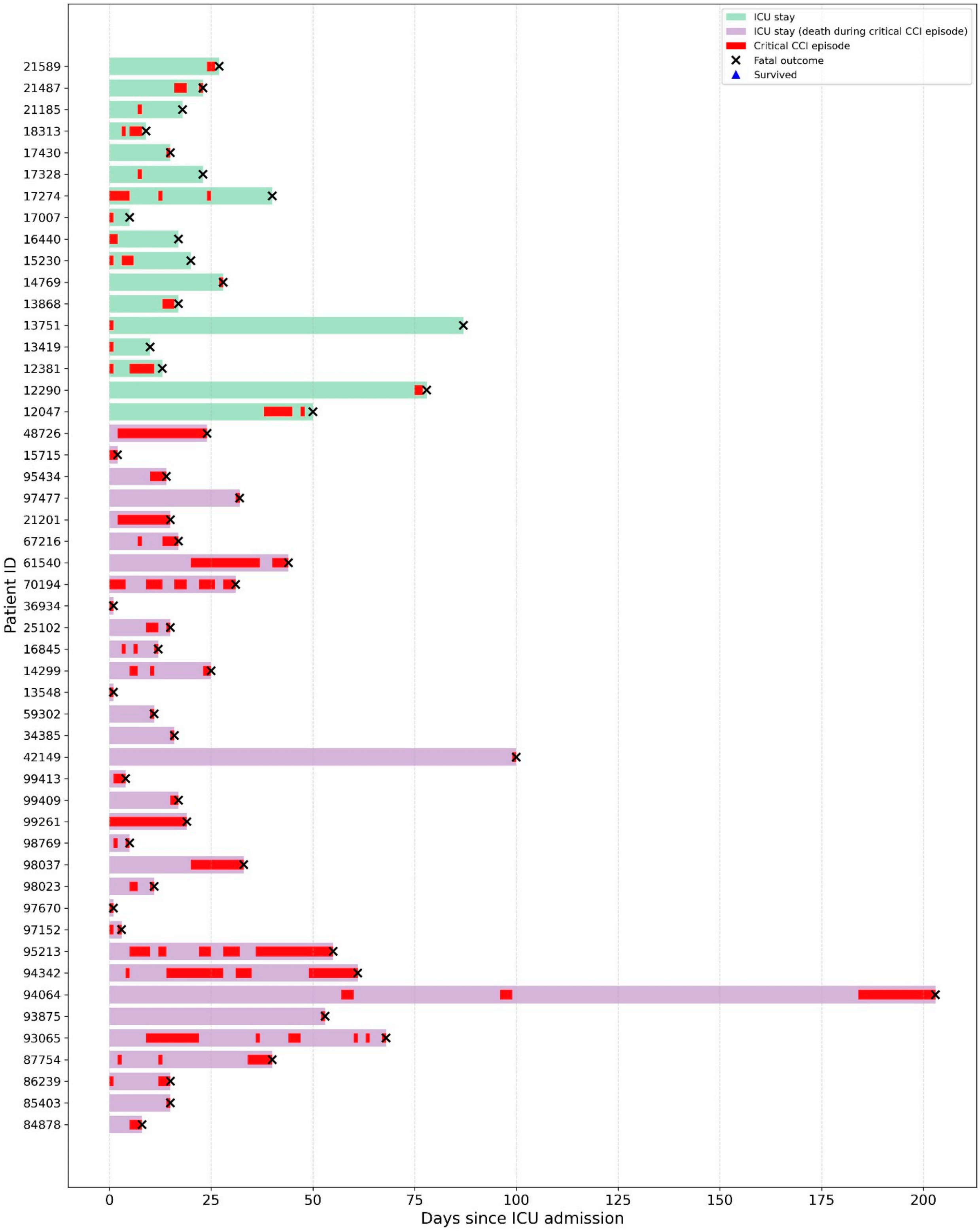

Supplemental figure S4. Swimmer plots of ICU stay and critical CCI episodes (part 4).

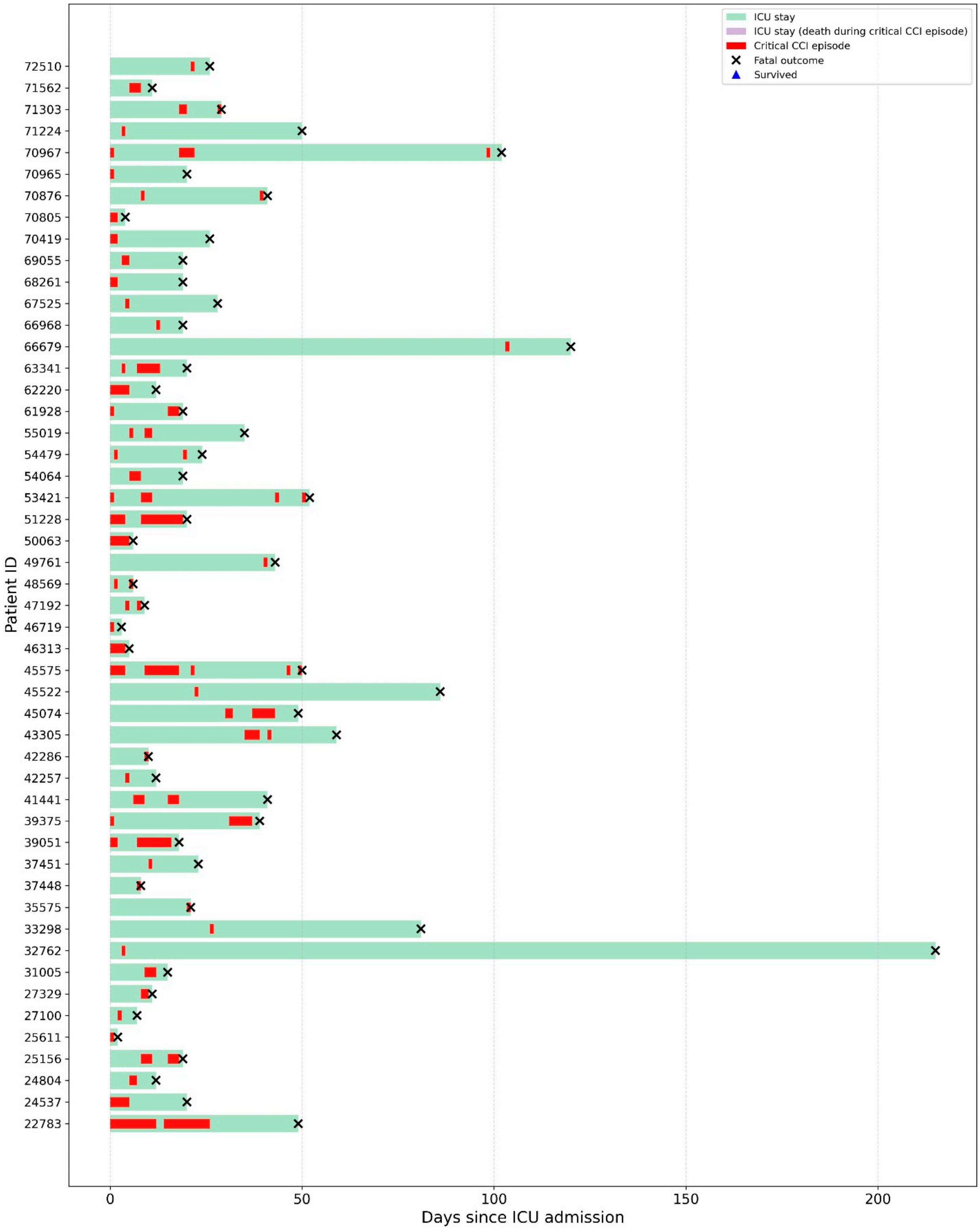

Supplemental figure S5. Swimmer plots of ICU stay and critical CCI episodes (part 5).

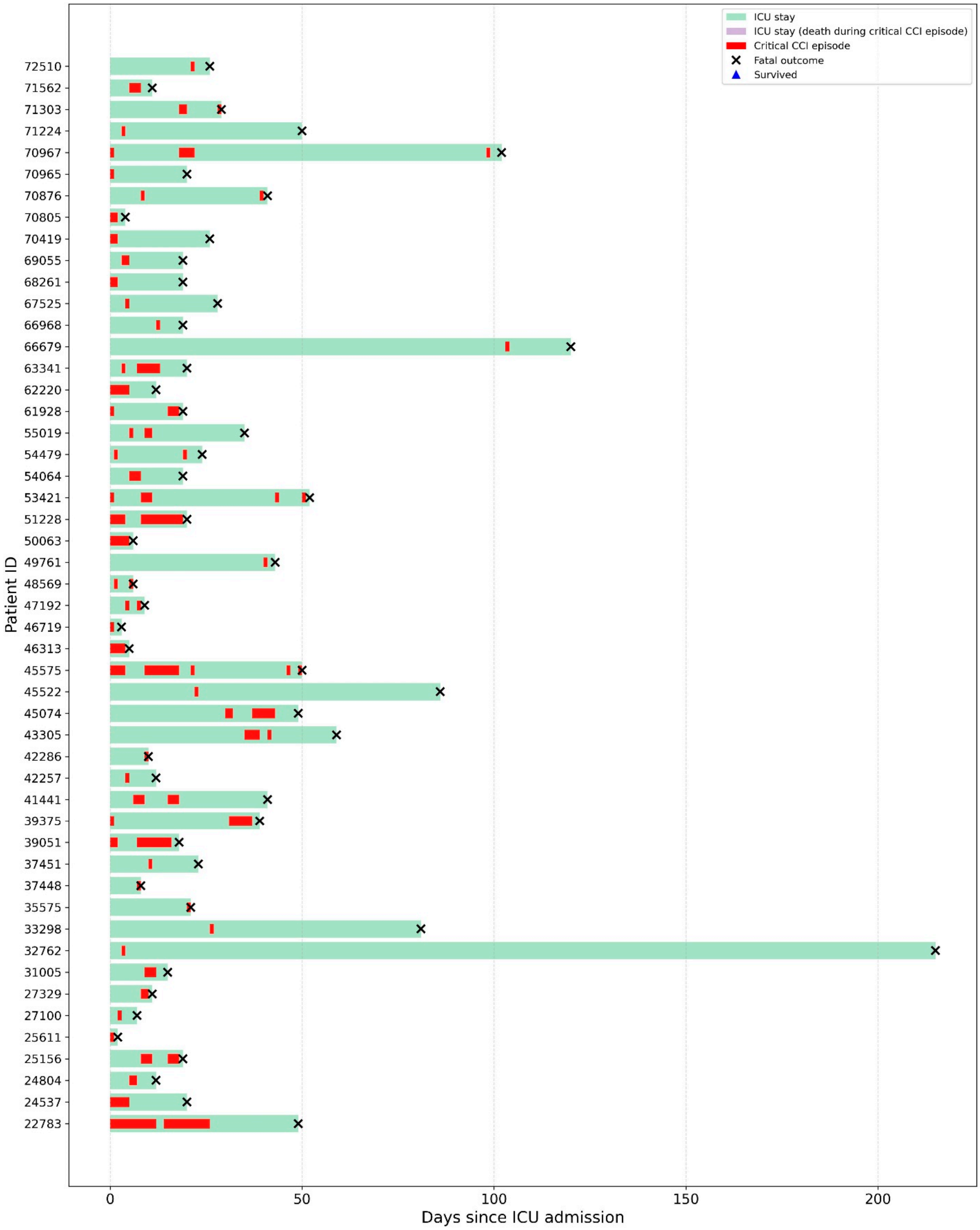

Supplemental figure S6. Swimmer plots of ICU stay and critical CCI episodes (part 6).

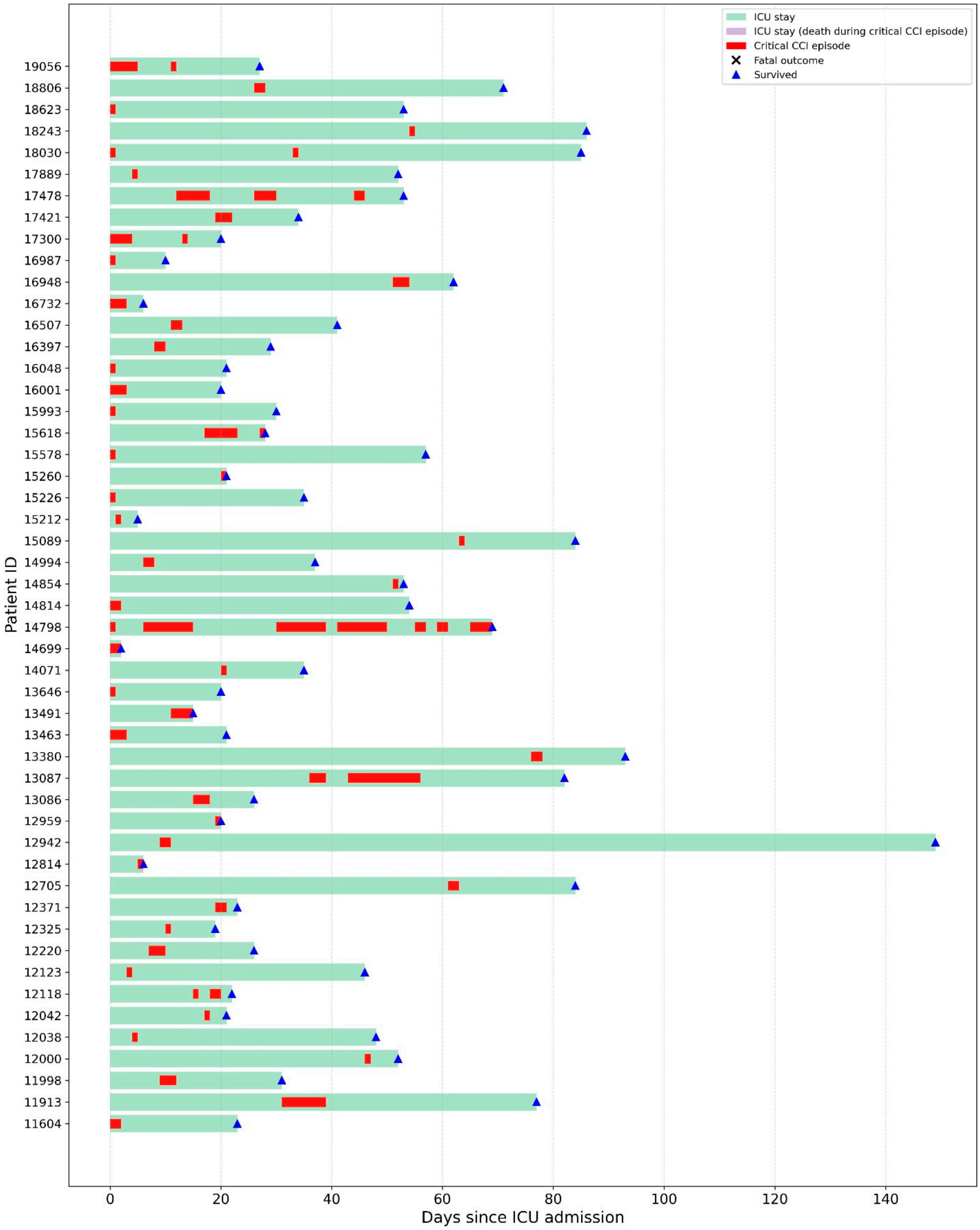

Supplemental figure S7. Swimmer plots of ICU stay and critical CCI episodes (part 7).

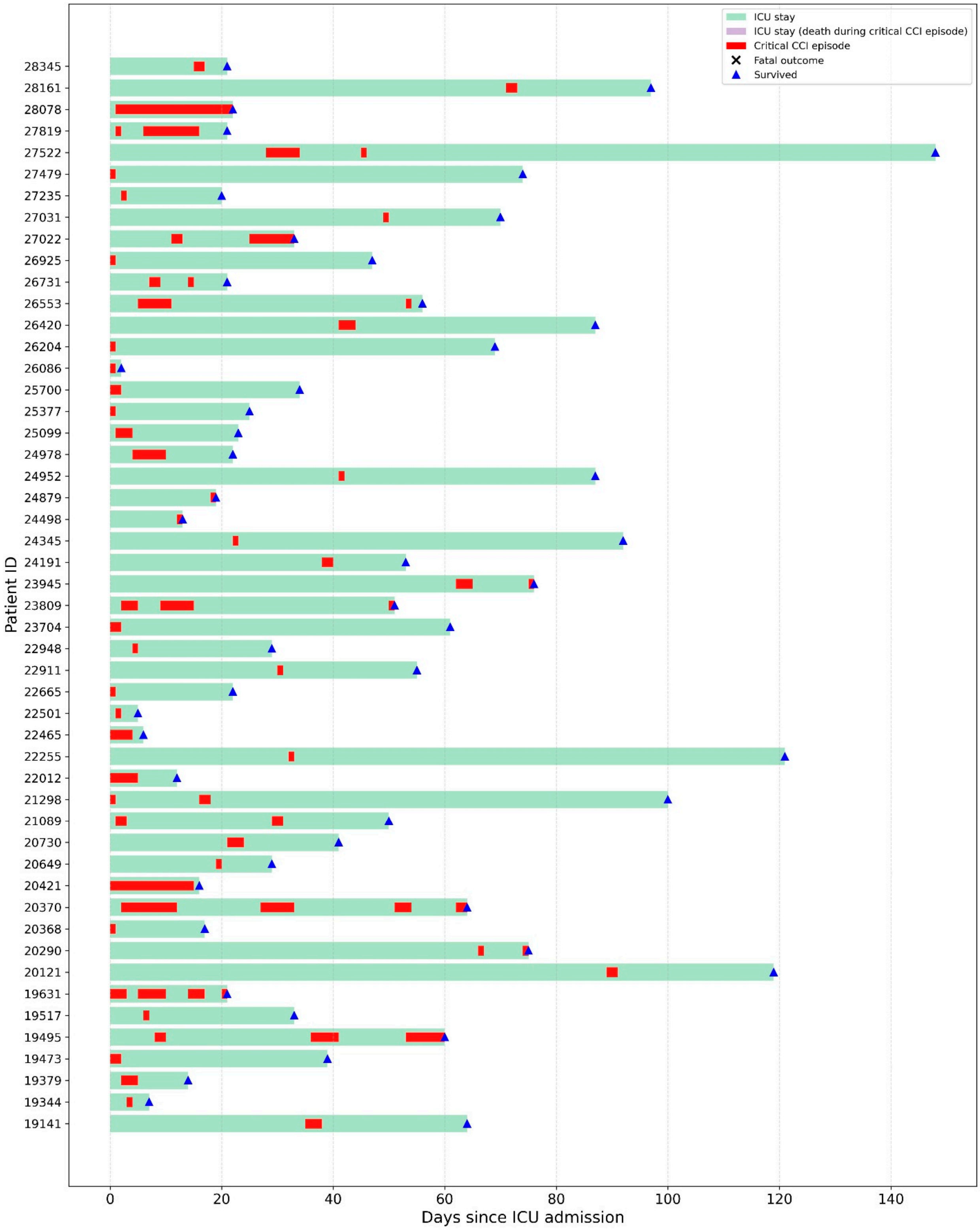

Supplemental figure S8. Swimmer plots of ICU stay and critical CCI episodes (part 8).

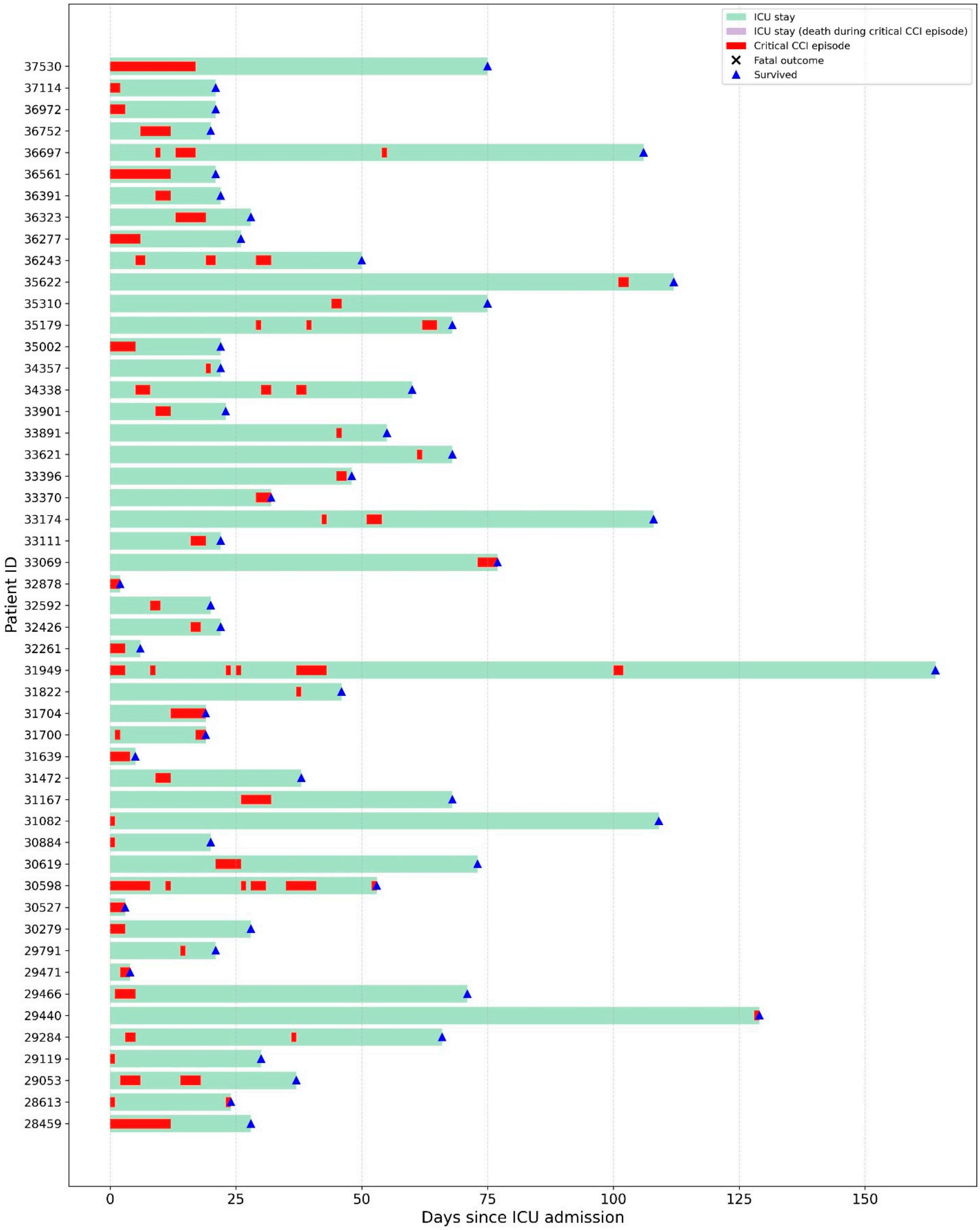

Supplemental figure S9. Swimmer plots of ICU stay and critical CCI episodes (part 9).

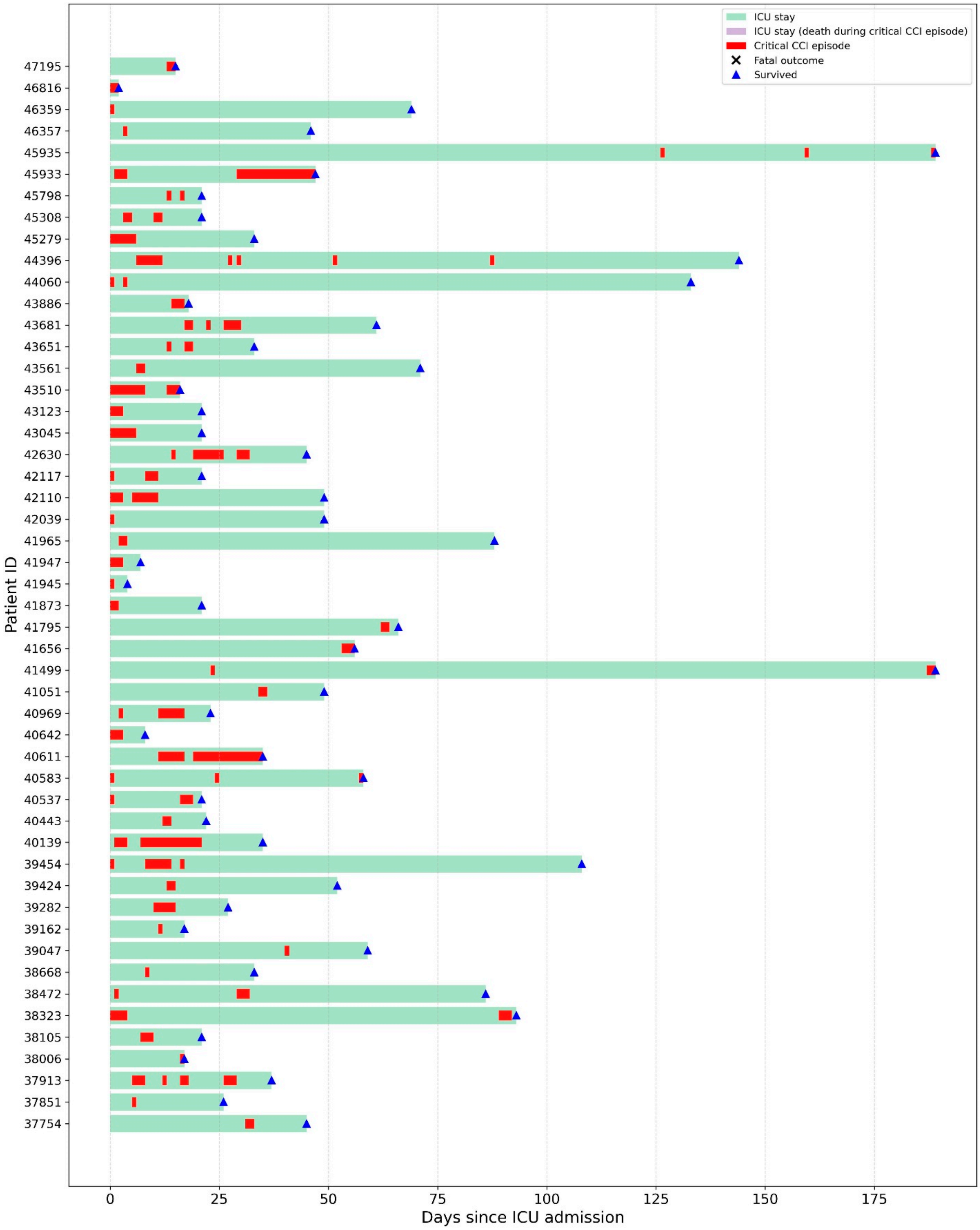

Supplemental figure S10. Swimmer plots of ICU stay and critical CCI episodes (part 10).

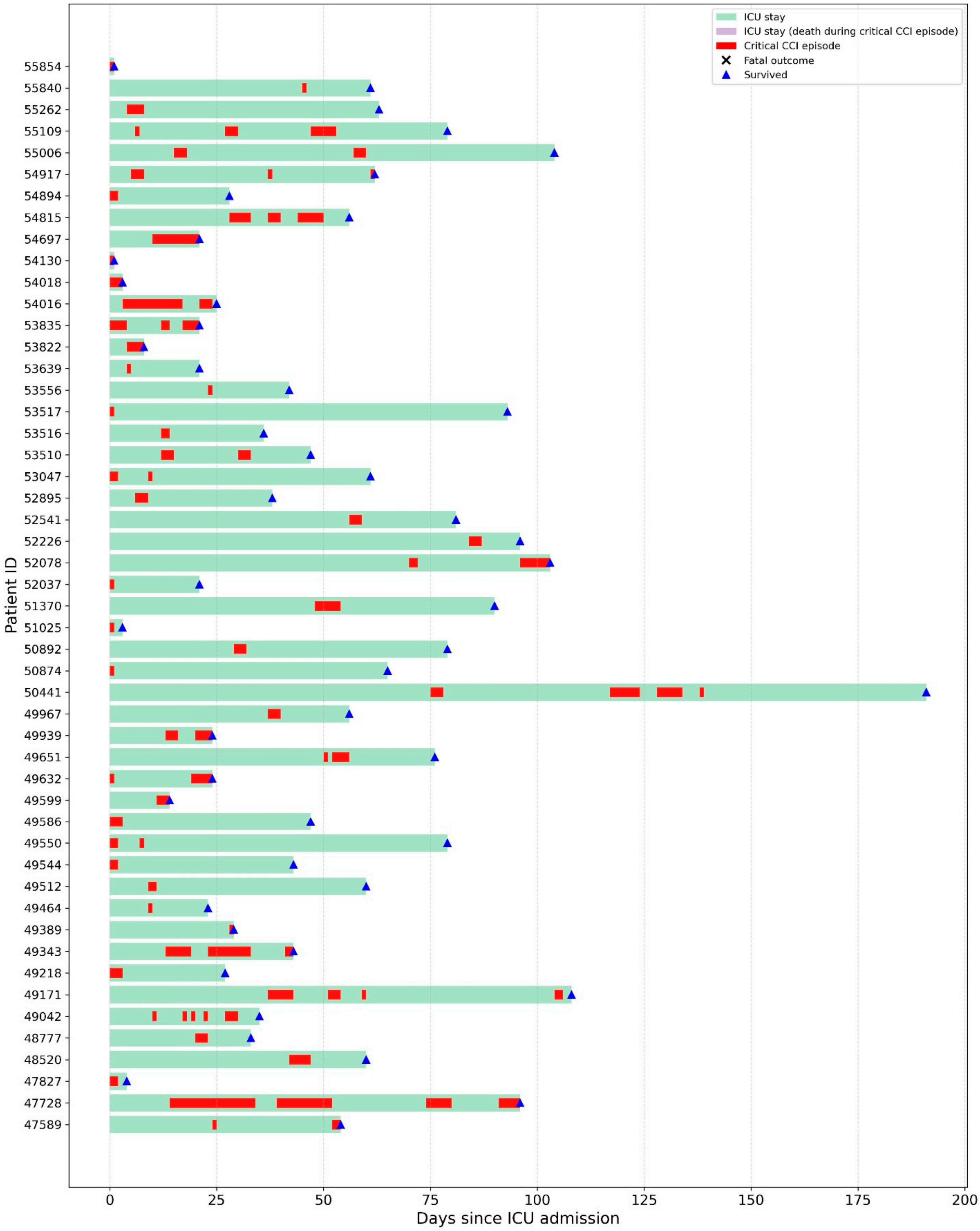

Supplemental figure S11. Swimmer plots of ICU stay and critical CCI episodes (part 11).

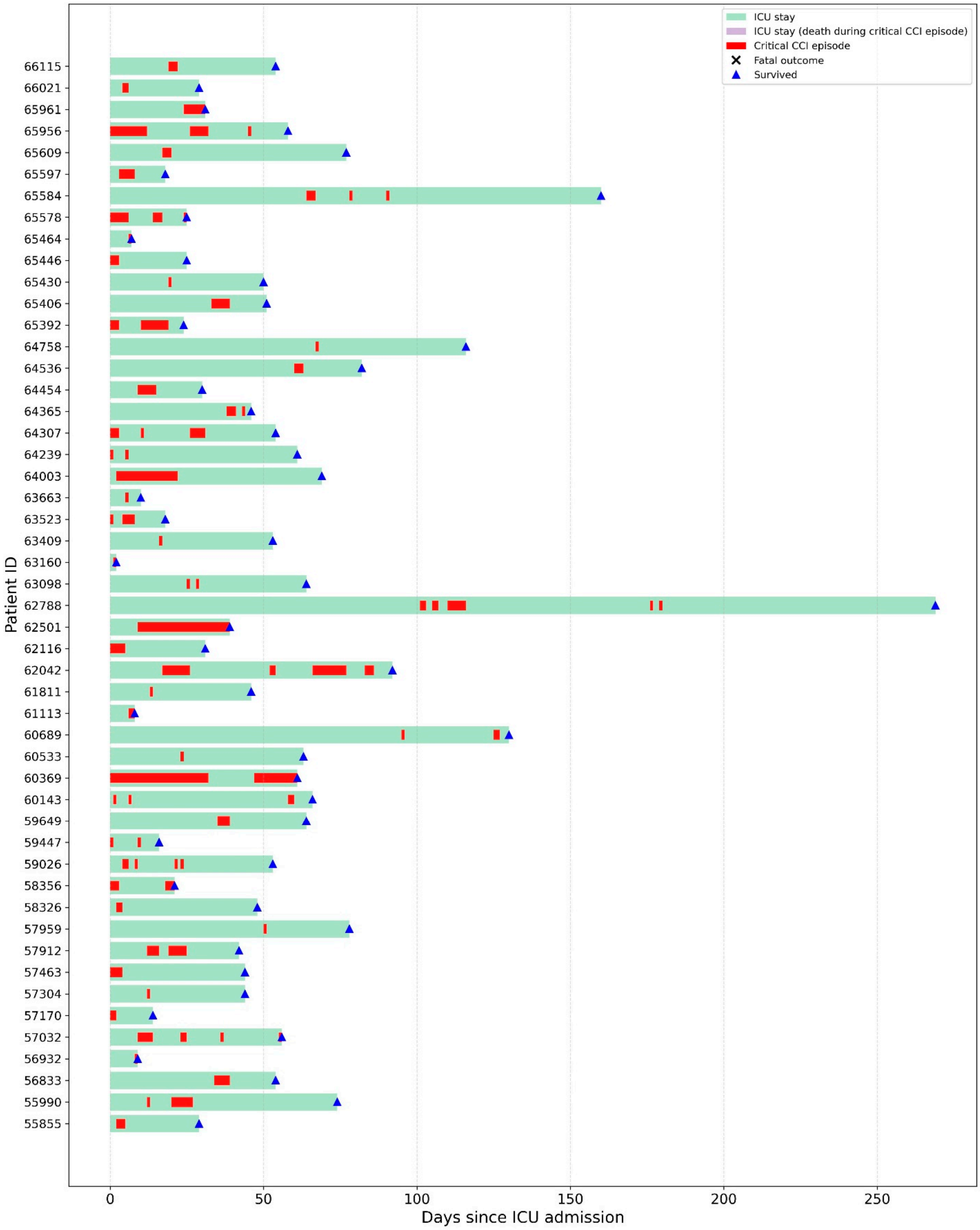

Supplemental figure S12. Swimmer plots of ICU stay and critical CCI episodes (part 12).

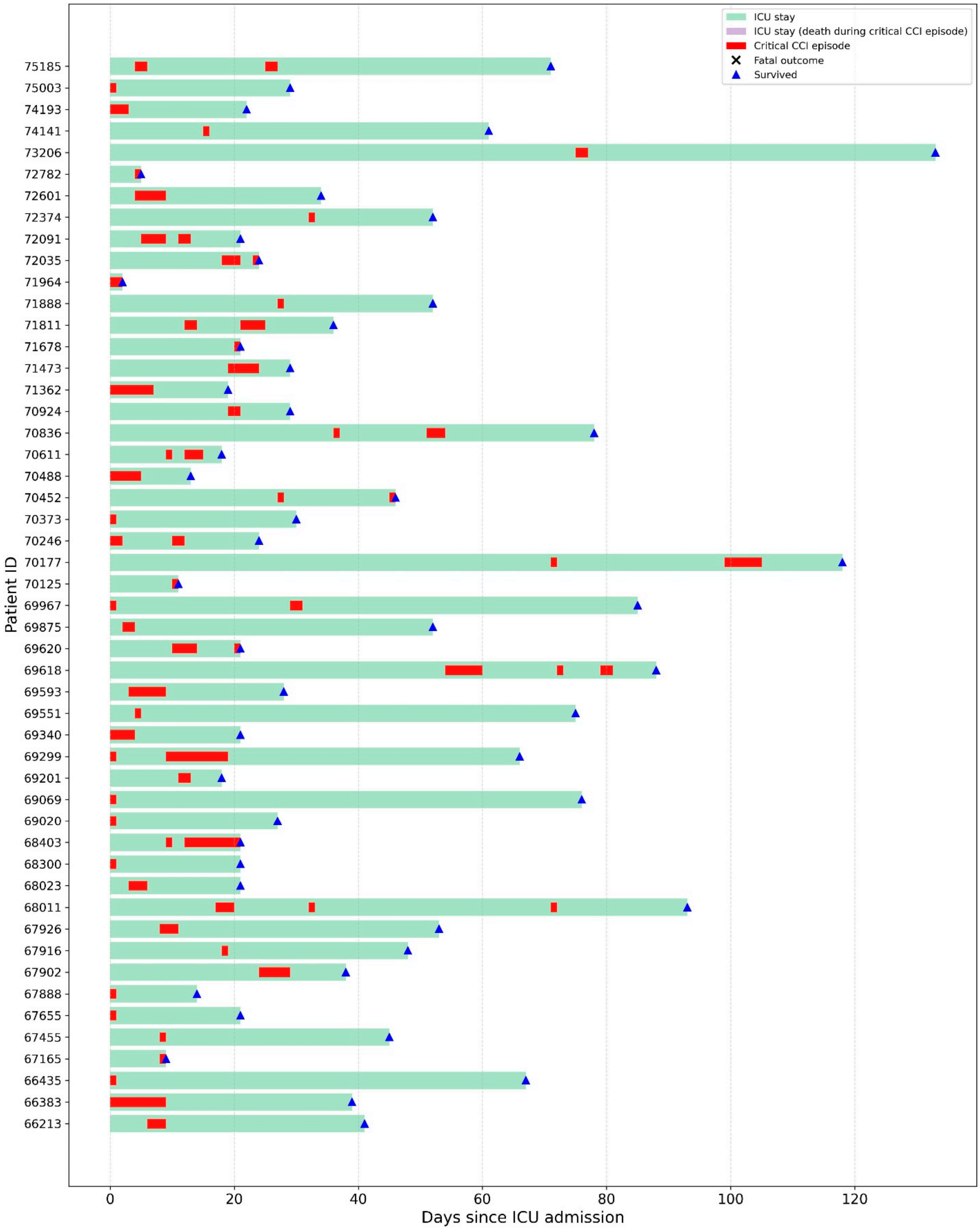

Supplemental figure S13. Swimmer plots of ICU stay and critical CCI episodes (part 13).

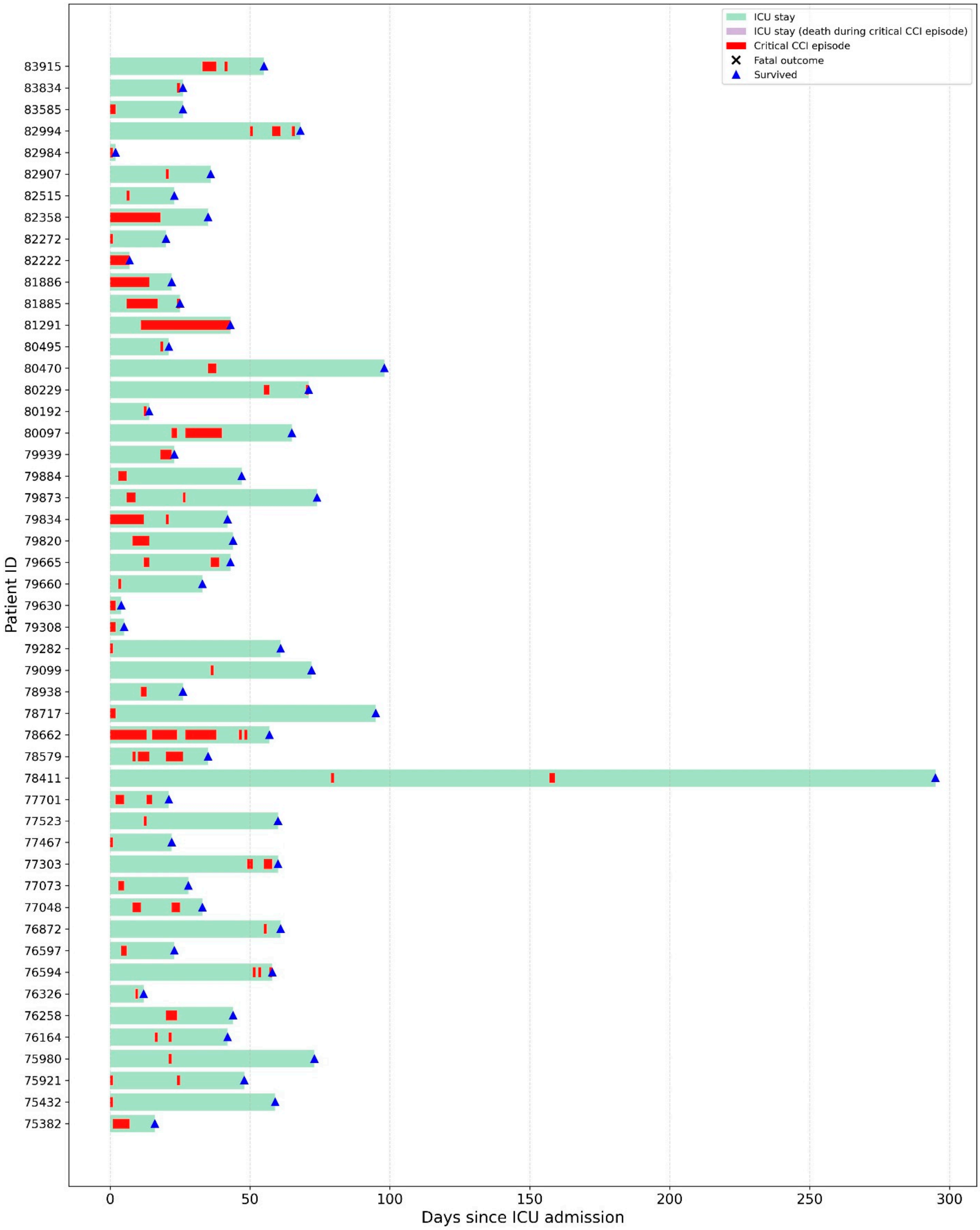

Supplemental figure S14. Swimmer plots of ICU stay and critical CCI episodes (part 14).

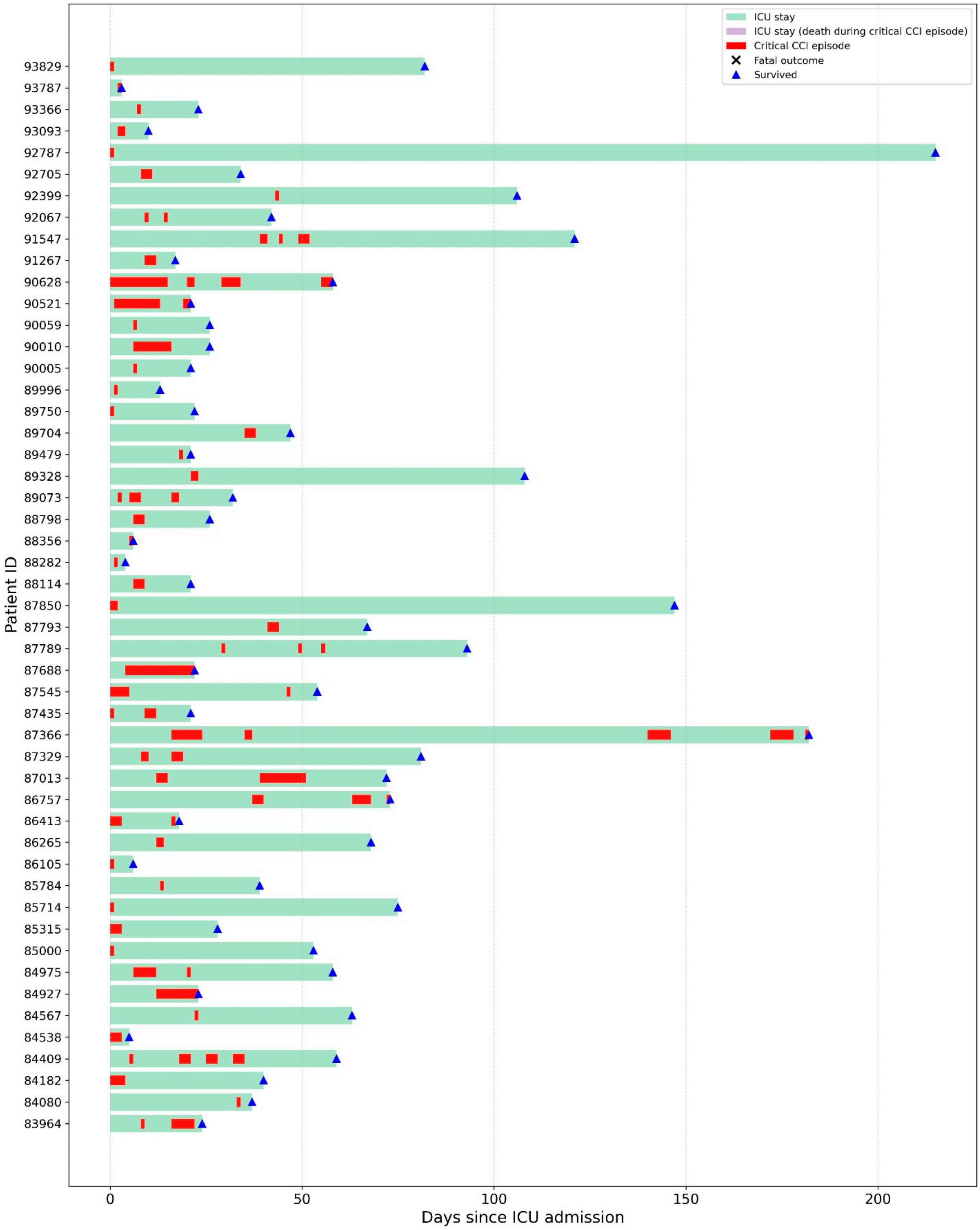

Supplemental figure S15. Swimmer plots of ICU stay and critical CCI episodes (part 15).

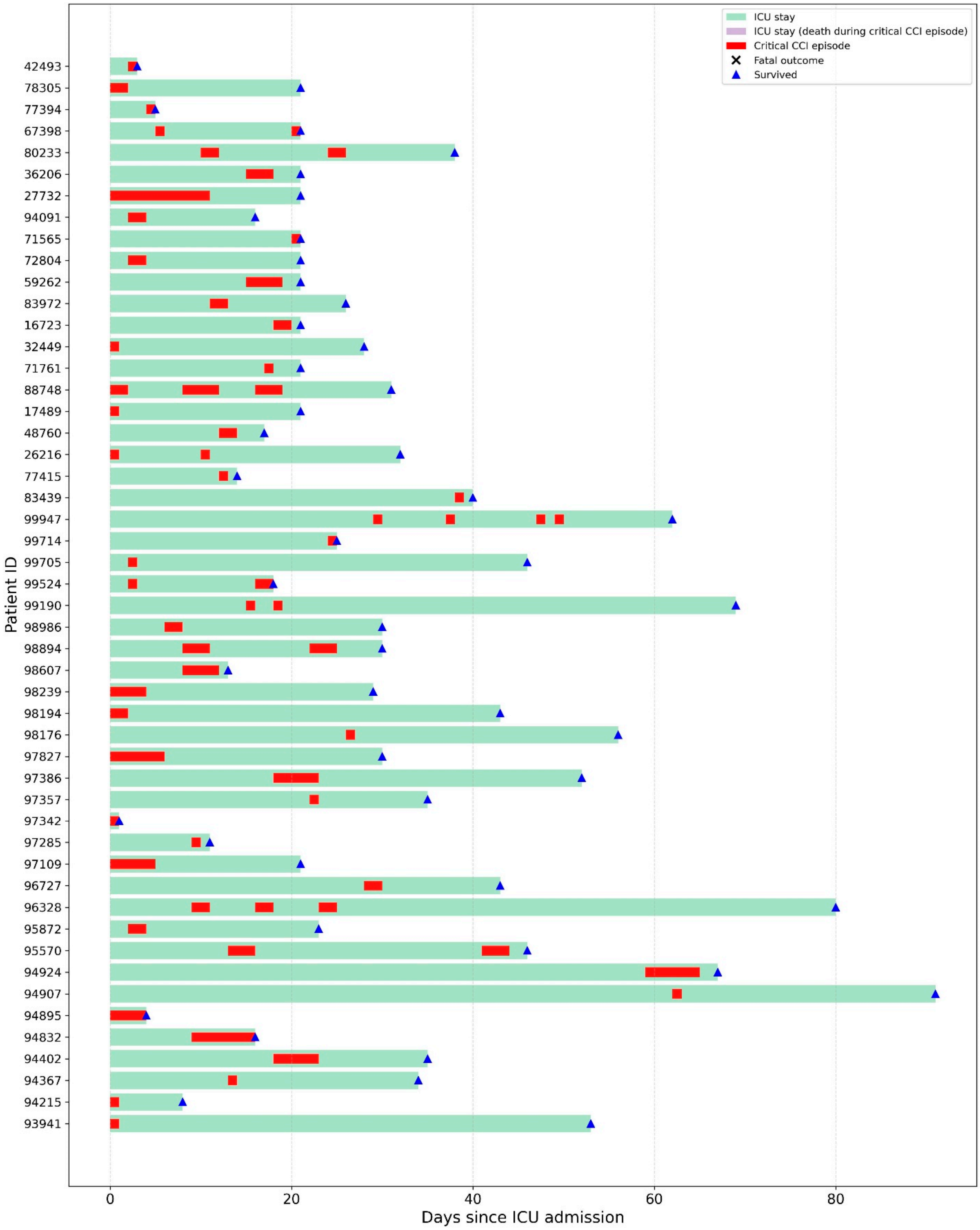

Supplemental figure S16. Swimmer plots of ICU stay and critical CCI episodes (part 16).

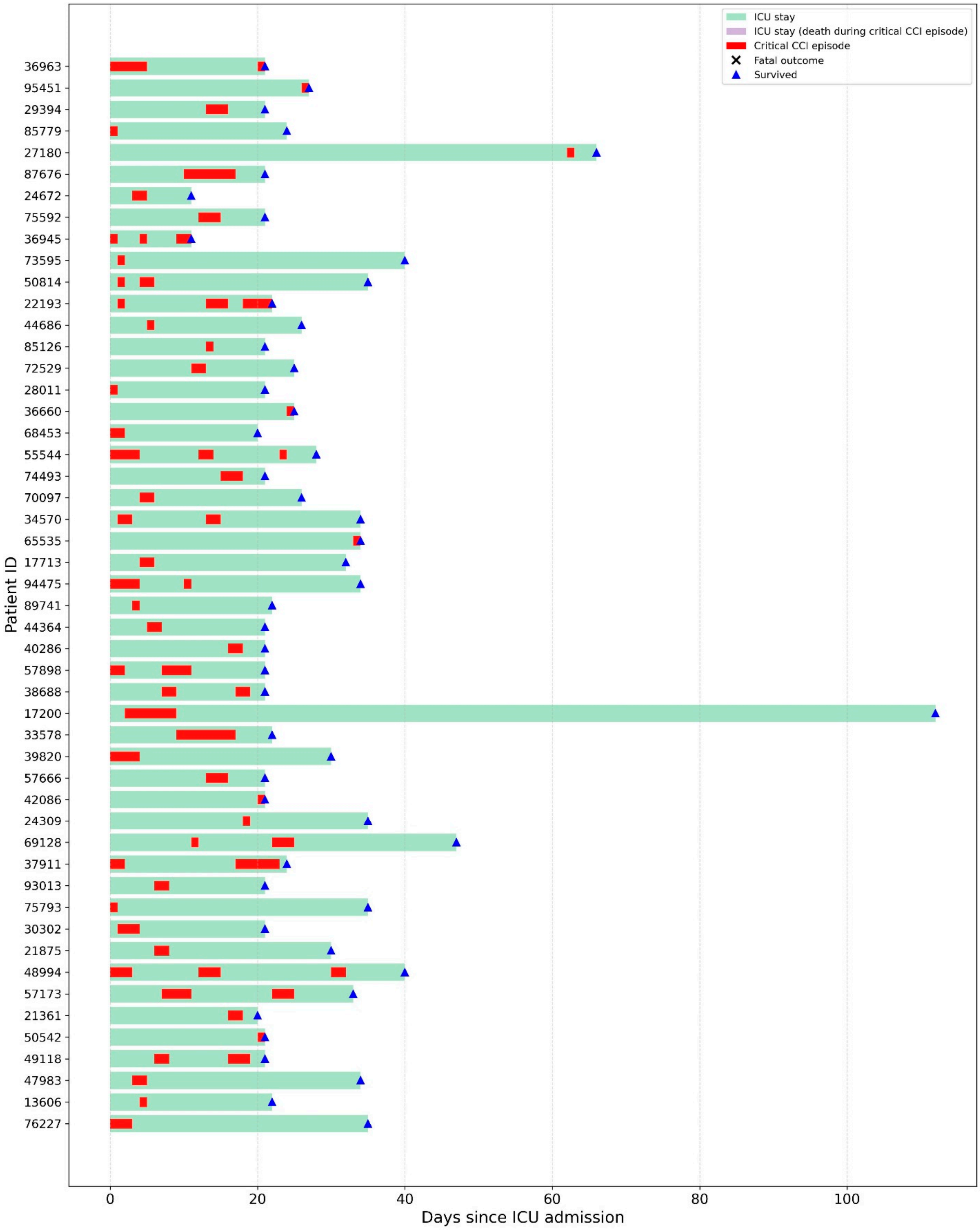

Supplemental figure S17. Swimmer plots of ICU stay and critical CCI episodes (part 17).

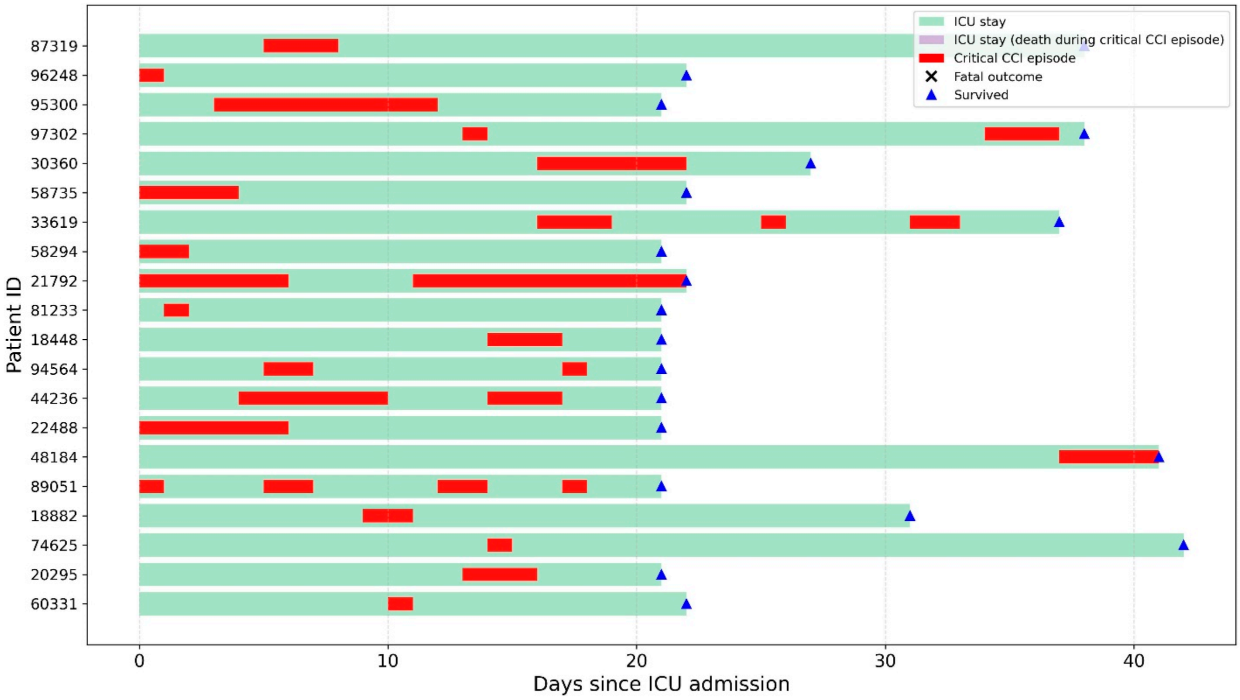

Supplement: Supplementary file 1 [file jcm-15-07864-s001.zip › jcm-4490614-supplementary.pdf]
